# Anticancer Effects of Fufang Yiliu Yin Formula on Colorectal Cancer Through Modulation of the PI3K/Akt Pathway and BCL-2 Family Proteins

**DOI:** 10.3389/fcell.2020.00704

**Published:** 2020-08-11

**Authors:** Bingzi Dong, Zhenjie Yang, Qiang Ju, Shigao Zhu, Yixiu Wang, Hao Zou, Chuandong Sun, Chengzhan Zhu

**Affiliations:** ^1^Shandong Key Laboratory of Digital Medicine and Computer Assisted Surgery, The Affiliated Hospital of Qingdao University, Qingdao, China; ^2^Department of Hepatobiliary and Pancreatic Surgery, The Affiliated Hospital of Qingdao University, Qingdao, China; ^3^Department of General Surgery, Anqiu People’s Hospital, Anqiu, China; ^4^Department of Blood Transfusion, The Affiliated Hospital of Qingdao University, Qingdao, China; ^5^Department of General Medicine, Weifang Hospital of Traditional Chinese Medicine, Weifang, China

**Keywords:** colorectal cancer, traditional Chinese medicine, network pharmacology, Fufang Yiliu Yin, BCL-2 family proteins

## Abstract

Colorectal cancer (CRC) is one of the most common malignant tumors in China. Fufang Yiliu Yin (FYY) is a traditional Chinese medicine formula used in clinical practice for cancer treatment, but its effectiveness and mechanism of action in human CRC are unclear. In this study, we investigated the antitumor effect of FYY on HCT116 and SW480 human CRC cell lines *in vitro* and evaluated the underlying molecular mechanism. A subcutaneous xenograft mouse model was used to confirm the antitumor effect *in vivo*. The components and targets of FYY were collected from the Traditional Chinese Medicine Systems Pharmacology Database (TCMSP) database. CRC targets were collected via the GeneCards and OMIM databases. Protein–protein interactions were explored using the STRING platform. Cytoscape was used to construct drug–disease–target networks. KEGG and GO analyses were performed to investigate common FYY and CRC targets. FYY significantly inhibited cell proliferation and induced HCT116 and SW480 cell apoptosis. Cell proliferation was blocked at the G0/G1 phase, while cell apoptosis was promoted at the early stage. According to the network pharmacological analysis, quercetin and kaempferol were the most bioactive compounds of FYY. The key targets of FYY were cyclin-D1, MAPK8, and EGFR. GO analysis showed that core targets included the apoptotic signaling pathway, response to steroid hormone, and cellular response to organic cyclic compound. The KEGG pathway analysis showed that FYY may affect CRC through the PI3K/Akt pathway. *In vitro*, FYY significantly inhibited tumor growth. Pathway analysis confirmed that FYY induced cell apoptosis by modulating PI3K/Akt signaling and BCL-2 family proteins. Hence, our findings indicate that FYY may be a promising adjuvant therapy for CRC.

## Introduction

Colorectal cancer (CRC) is one of the most common malignant tumors in China (Chen et al., 2016). CRC is one of the five leading causes of cancer death, and its incidence is gradually increasing owing to obesity and lifestyle changes (Du et al., 2015; Chen et al., 2016). Postoperative treatments including chemotherapy and radiotherapy are important for longer patient survival. Traditional Chinese medicine (TCM) has become an option for preventing CRC metastasis and enhancing the effects of chemotherapy (Shi et al., 2017; Xu et al., 2017; Xie et al., 2019). TCM is used as an alternative or supplementary treatment in the United States and Europe and has been widely used to treat various diseases in Asia, especially in China (Wang et al., 2014). TCM has also been widely investigated in Asia for effective and low-toxicity monomer compounds to develop new drugs for cancer therapy and to counteract drug resistance (Sui et al., 2017; Zheng et al., 2018; Xie et al., 2019).

In China, patients usually choose TCM for adjuvant therapy after curative resection (Xu et al., 2017). The effectiveness of TCM has been proven in multiple cancers including breast cancer (Lee et al., 2014), hepatocellular carcinoma (Chen et al., 2018), pancreatic cancer (Kuo et al., 2018), and CRC (Shi et al., 2017; Xu et al., 2017). In CRC, TCM significantly improved disease-free survival in stage II and III CRC in a retrospective cohort study including 817 patients (Shi et al., 2017). In a multicenter prospective cohort study including 312 patients with stage II and III CRC, postoperative TCM treatment was associated with better disease-free survival and overall survival compared to those of the untreated group (Xu et al., 2017). Certain active ingredients in TCM herbs may have stronger activity in inhibiting cell proliferation and promoting cell apoptosis (Tan et al., 2011; Huang and Hu, 2018). For example, bufalin, an active component of the TCM Chan Su, can reverse multidrug resistance by inhibiting the protein expression and efflux function of ABCB1 (Yuan et al., 2017). Cinobufagin, another cardiotonic steroid isolated from Chan Su, suppresses tumor neovascularization by disrupting the endothelial mTOR/HIF-1α pathway to trigger reactive oxygen species-mediated vascular endothelial cell apoptosis (Li et al., 2019). Of the frequently used TCM treatments, the most effective single herbs are Ginseng Radix (Ren Shen), *Hedyotis diffusa* Willd (Bai Hua She She Cao), *Scutellaria barbata* (Ban Zhi Lian), and Astragali Radix (Huang Qi) (Lee et al., 2014; Wu et al., 2017). However, the underlying mechanisms of these remedies remain unknown. Network pharmacology can efficiently and quickly identify the interactions between drugs and target proteins, providing a foundation for TCM application (Zhang et al., 2019).

Fufang Yiliu Yin (FYY) is a TCM formula that has been used in clinical practice for cancer treatment. Our previous study found that FYY inhibited cell proliferation, migration, and invasion and promoted apoptosis in hepatocellular carcinoma (Yang et al., 2018). FYY contains eight herbs: Astragali Radix (Huang Qi), *Ganoderma lucidum* (Ling Zhi), Semen Armeniacae Amarum (Ku Xing Ren), *H. diffusa* Willd (Bai Hua She She Cao), Aconiti Lateralis Radix Praeparata (Fu Zi), *Glycyrrhiza glabra* Linne (Gan Cao), Radix Panacis Quinquefolii (Xi Yang Shen), and Platycodi Radix (Jie Geng). Of these herbs, Radix Panacis Quinquefolii (Ginseng Radix), *H. diffusa* Willd, and Astragali Radix are commonly used in anticancer formulas (Lee et al., 2014; Wu et al., 2017). *G. lucidum* and Platycodi Radix also reportedly have anticancer effects; Radix Astragali (Jung et al., 2016), *G. lucidum* (Dai et al., 2014), Platycodi Radix (Park and Lee, 2014), and *H. diffusa* Willd (Zhang et al., 2016) inhibit cancer cell proliferation. Polysaccharides in *G. lucidum* inhibit the proliferation of CRC cells, upregulating the expression of P21 protein and blocking cells at the G2/M phase (Na et al., 2017).

In the current study, we investigated the anticancer effect of FYY on CRC cells *in vitro* and *in vivo*, and a network pharmacology analysis was performed to explore the potential molecular mechanisms. The information obtained in this study will aid in elucidating the previously unavailable mechanisms of action of FYY in CRC and developing FYY as an adjuvant therapy for CRC.

## Materials and Methods

### Preparation of FYY and Cell Culture

The components of FYY conformed to the provisions stated by the Chinese Pharmacopoeia; FYY was prepared at the Weifang Hospital of Traditional Chinese Medicine, Shandong, China (Yang et al., 2018). FYY (120 mg/ml) was stored at −20°C until use and was further diluted to the required concentrations in subsequent cell experiments. Human CRC cell lines (HCT116 and SW480) were purchased from the cell resource center of the Shanghai Institutes for Biological Sciences, Chinese Academy of Sciences (Shanghai, China). HCT116 cells were grown in RPMI-1640 medium (RPMI-1640, HyClone, United States) and SW480 cells were grown in Dulbecco’s modified Eagle’s medium (DMEM, HyClone, United States) containing 10% fetal bovine serum (FBS, Gibco BRL, United States) and 1% penicillin/streptomycin (Sigma-Aldrich; St. Louis, MO, United States) in 5% CO_2_ at 37°C in a humidified incubator.

### Cell Viability and Colony Formation Assays

Cells (3 × 10^3^ per well) were seeded into 96-well plates and incubated overnight at 37°C, 5% CO_2_ in a humidified incubator. When the cells adhered to the wall, HCT116 and SW480 cells were treated with 3, 6, 9, 12, or 15 mg/ml of FYY or PBS as a control for 24 and 48 h. Cell viability was measured using a cell counting kit-8 (CCK-8, Beyotime Institute of Biotechnology, Inc., Shanghai, China). Ten microliters of the CCK-8 solution was added to each well, and then samples were incubated at 37°C for 2 h. Finally, the absorbance value at 450 nm was determined using a Multiskan^TM^ FC Microplate Photometer (Thermo Fisher Scientific Inc., United States).

HCT116 and SW480 cells were treated with 9, 12, or 15 mg/ml of FYY or PBS as a control for 24 h. The cells (1 × 10^3^ per well) were then cultured in six-well plates, and the medium was changed every 3 days for 14 days. Cell colonies were fixed with 4% paraformaldehyde and then stained with 5% Giemsa (Beyotime Institute of Biotechnology, Inc., Shanghai, China) for 15 min. A colony formation assay was performed to count viable colonies (>50 cells per colony).

### Cell Cycle Analyses

HCT116 and SW480 cells were treated with 9, 12, or 15 mg/ml FYY or PBS as a control for 24 h. The collected cells (1 × 10^6^) were fixed in cold ethanol and stored at 4°C overnight. The next day, the cells were washed twice with cold PBS; then 100 μl RNase A (25 μg/ml) and 400 μl propidium iodide (50 μg/ml, Sigma Aldrich; St. Louis, MO, United States) were added to each sample and incubated for 30 min in the dark. Measurements were taken using a flow cytometer (FACScan; BD Biosciences, Bedford, MA, United States), and the data were analyzed using FlowJo 7.6 software (Tree Star, Inc., Ashland, OR, United States).

### Cell Apoptosis Analyses

Cell apoptosis was detected using an Apoptosis-Hoechst 33258 Staining Kit (Beyotime Biotechnology, Shanghai, China). Samples were fixed with 4% paraformaldehyde at room temperature for 10 min and stained with 10 mg/ml Hoechst 33258 at room temperature for 15 min. Then, fluorescence was detected under an Olympus IX50 microscope (Olympus Corp., Tokyo, Japan) at × 400 magnification. Apoptotic cells were identified using an Alexa Fluor 488 Annexin V/Dead Cell Apoptosis Kit (Invitrogen^TM^/Molecular Probes, Eugene, OR, United States). After centrifugation at 300 *g* for 5 min, the cell density was counted and diluted in 1 × annexin-binding buffer to obtain 1 × 10^6^ cells/ml (100 μl per assay). Cells were stained with 5 μl of annexin V-FITC and 1 μl propidium iodide at room temperature for 20 min in the dark, and then 400 μl of binding buffer was added. Measurements were taken using a flow cytometer, and the data were analyzed using FlowJo 7.6 software.

### Network Pharmacology

Active FYY compounds were screened using the Traditional Chinese Medicine Systems Pharmacology Database (TCMSP)^1^ (Ru et al., 2014). With the pharmacokinetic information retrieval filter based on the TCMSP platform, the oral bioavailability and drug-likeness were set to ≥30% and ≥0.18 to obtain qualified herbal compounds. The chemical structures of the compounds were drawn using ChemBioOffice 2010 (Kerwin, 2010). CRC targets were predicted and screened using the GeneCards database^2^ (Stelzer et al., 2016) and OMIM platform^3^ (Amberger and Hamosh, 2017). Venny 2.1.0^4^ (Venny, 2018) was used to screen for common targets between FYY and disease-related targets.

Drug compound–disease–target networks were built using Cytoscape (v 3.7.1) software (Shannon et al., 2003), and the merge function was used to analyze the core compounds. Protein interaction networks of the common FYY and CRC-related targets were built using the String database platform with medium confidence (0.7) and rejecting the target protein independent of the network (Szklarczyk et al., 2017).

Gene ontology (GO) analysis and Kyoto Encyclopedia of Genes and Genomes (KEGG) pathway analysis were performed using Metascape (Zhou et al., 2019). Enriched GO terms and relevant pathways with *p*-values < 0.05 were selected for better prediction and verification of the biological process and mechanism.

### Western Blot Analysis

The following primary antibodies obtained from Cell Signaling Technology Inc. (Danvers, MA, United States) were used in the immunoblotting analysis: PI3K (p110α, #4255, 1:1,000), AKT (pan, #2920, 1:1,000), p-AKT (Ser473, #4060, 1:1,000), BCL-2 (#4223, 1:1,000), BCL-XL (#2762, 1:1,000), BAX (#5023, 1:1,000), P21 (#2947, 1:1,000), C-MYC (#18583, 1:1,000), and GAPDH (#5174, 1:1,000). Total proteins were extracted from cells and tissues using RIPA lysis buffer (CWBIO, Beijing, China). Equal amounts of protein from each sample were separated by 10% SDS-PAGE electrophoresis and then transferred onto 0.45-μm PVDF membranes (Bio-Rad Laboratories, Hercules, CA, United States). Subsequently, the membranes were blocked with 5% milk in PBS plus 0.1% Tween 20 (PBST) for 60 min, incubated with primary antibodies overnight at 4°C, and then incubated with goat anti-rabbit horseradish peroxidases (Abcam, Cambridge, MA, United States; 1:10,000) or goat anti-mouse horseradish peroxidases (Abcam, Cambridge, MA, United States; 1:10,000) for 2 h at room temperature. Finally, the band was detected using an enhanced chemiluminescence reagent and visualized with a Fusion FX7 System (Vilber Lourmat, France). ImageJ software was used to calculate the intensity (gray value) of each protein band, and GAPDH served as a control for normalization.

### Tumor Xenografts in Nude Mice

Ten male BALB/c nude mice (4–5 weeks old, 20.2 ± 1.9 g) were purchased from Beijing Vital River Laboratory Animal Technology Co. Ltd. (Beijing, China). The mice were housed at 24 ± 1°C under a 12-h light/dark cycle with free access to food and water. All animal experiments were completed at the specific-pathogen-free medical animal laboratory of The Affiliated Hospital of Qingdao University and approved by the Animal Ethics Committee of The Affiliated Hospital of Qingdao University (AHQU20180310A). HCT116 cells (1 × 10^7^ cells per tumor) were subcutaneously injected into the right armpit of the nude mice. Seven days after tumor inoculation, the tumor size was measured using a Vernier caliper, and the mice were divided into two groups: the FYY treatment group and a control group (*n* = 5 mice per group). The FYY group was intragastrically administered 0.2 ml/10 g body weight daily in a primary concentration of 120 mg/ml. The control group was intragastrically administered an equivalent volume of PBS. Tumor sizes were measured every 3 days and calculated using the following formula: tumor volume (mm^3^) = 0.5 × length × width^2^. The nude mice were killed by cervical dislocation on day 36, and the tumors were excised, weighed, and photographed. Finally, tumor tissue and liver tissue were stored in 10% formalin or at −80°C for subsequent immunohistochemistry or western blot analyses, respectively.

### Immunohistochemistry

Tumor and liver tissues of the nude mice were fixed with 10% paraformaldehyde for 12 h and then embedded in paraffin. Embedded paraffin sections were de-waxed in xylene and rehydrated in ethanol. Antigen retrieval was performed in 0.01 M citrate buffer (pH 6.0) using a pressure cooker followed by incubation for 3 min. Samples were then washed thrice with PBS and fixed in 95% ethanol for 30 min. Ki-67 antibody (Novus, Colorado, United States; 1:600) was stained with a streptavidin–peroxidase detection kit (ZSGB-BIO, Beijing, China) according to the kit instructions.

### Statistical Analysis

Data analysis was performed using GraphPad Prism 6.0 software (San Diego, CA, United States). All experimental data were expressed as the mean ± SD. The statistical significance of the results was analyzed by one-way analysis of variance (ANOVA) for multiple group comparisons and Student’s *t*-test for two group comparisons. A value of *p* < 0.05 was considered statistically significant. All experiments were performed in triplicate.

## Results

### FYY Inhibited Proliferation and Promoted Apoptosis of CRC Cells *in vitro*

**FIGURE 1:**
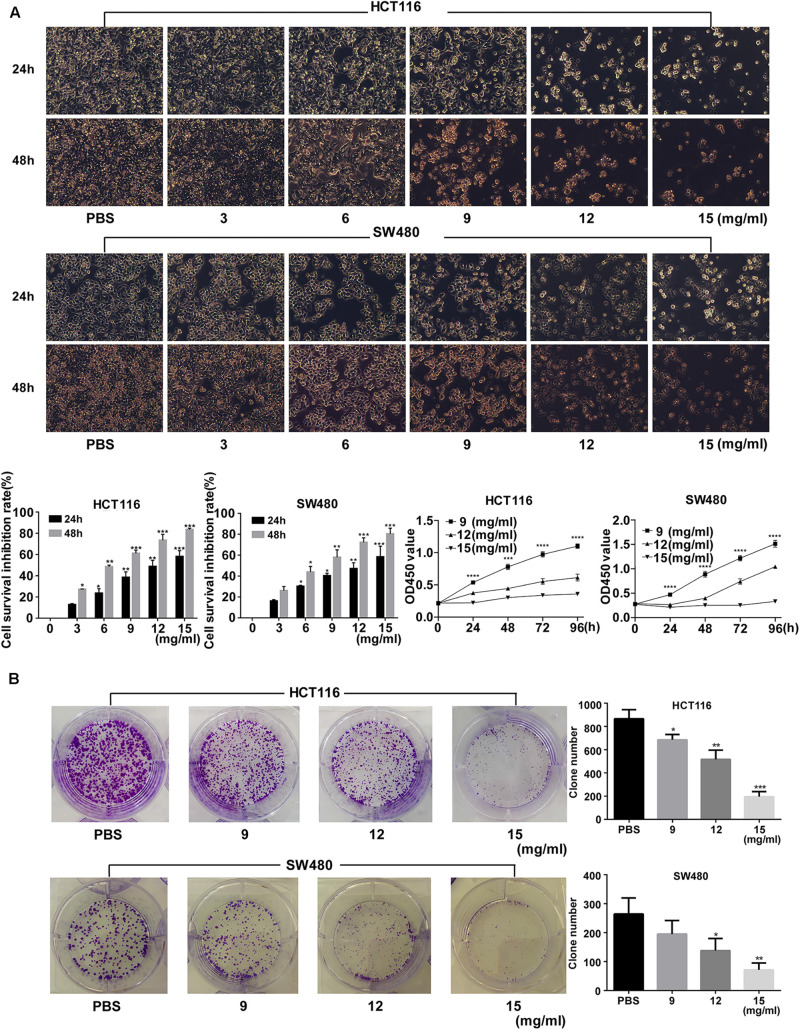
Fufang Yiliu Yin (FYY) inhibited colorectal cancer cell proliferation. **(A)** CCK-8 assay indicated that FYY inhibited the proliferation of HCT116 and SW480 cells in a dose- and time-dependent manner after 24 and 48 h of treatment. PBS was used for the control treatment (*n* = 6 per group). **(B)** Colony formation ability decreased after treatment with different concentrations of FYY for both HCT116 and SW480 (*n* = 3 per group). Values are shown as the mean ± SD, *p < 0.05, **p < 0.01, and ***p < 0.001 vs. control group. The *p*-values were obtained using ANOVA.

Fufang Yiliu Yin significantly inhibited the growth of HCT116 and SW480 cells in a dose-dependent manner (Figure 1A). The colony formation assay showed that the number of the colonies in the FYY group (12 and 15 mg/ml) was lower than that of the control group (Figure 1B).

**FIGURE 2:**
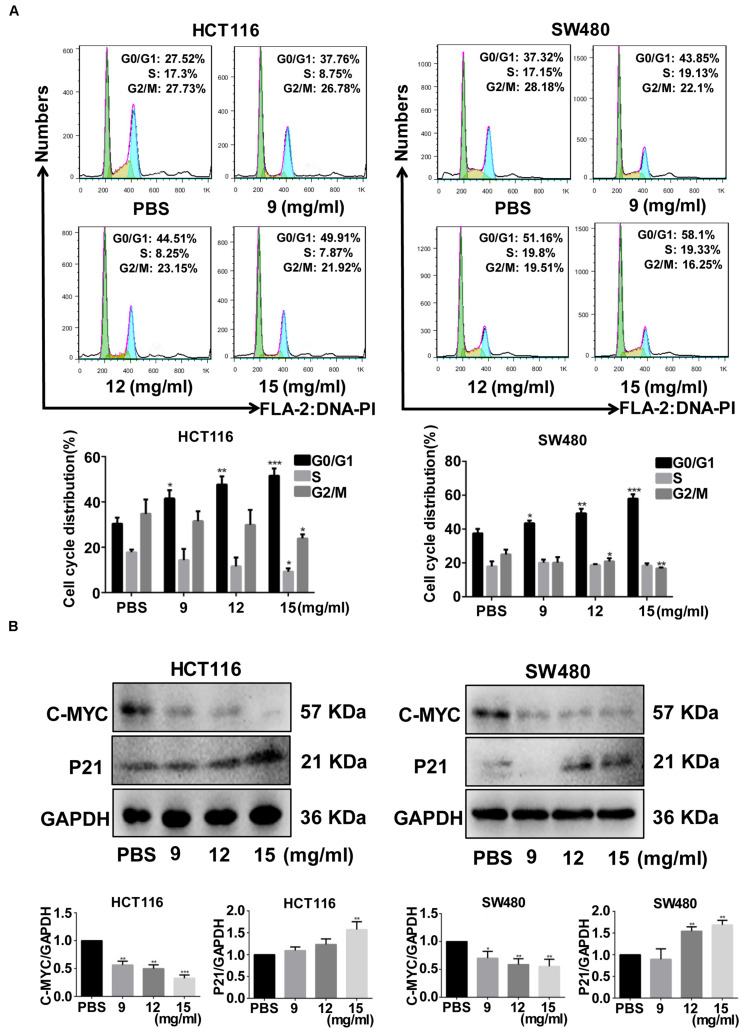
Fufang Yiliu Yin (FYY) significantly inhibited the colorectal cancer cell cycle. **(A)** FYY significantly inhibited the cell cycle progress of HCT116 and SW480, arresting them at the G2/M phase as shown by flow cytometry assay (*n* = 3 per group). **(B)** The expression of C-MYC decreased and P21 increased with FYY treatment (*n* = 3 per group). Values are shown as the mean ± SD, *p < 0.05, **p < 0.01, and ***p < 0.001 vs. control group. The *p*-values were obtained using ANOVA.

Colony formation ability was significantly inhibited by >9 mg/ml (*p* = 0.035) and >12 mg/ml (*p* = 0.030) FYY for HCT116 and for SW480 cells, respectively. The cell cycle analysis showed no significant difference in the percentage of cells in S (*p* = 0.584 for 9 mg/ml) and G2/M phases (*p* = 0.864 for 9 mg/ml) in HCT116. However, a significant increase in G0/G1 phase was found after treatment with increasing concentrations of FYY (*p* = 0.013 for 9 mg/ml, Figure 2A) in HCT116. Similar results were obtained for SW480 cells. FYY blocked cell cycle at the G0/G1 phase in a concentration-dependent manner. FYY inhibited the expression of C-MYC (*p* < 0.001 for 9 mg/ml) and promoted the expression of P21 protein (*p* < 0.001 for 15 mg/ml, Figure 2B) in HCT116; similar results were observed in SW480 cells. This indicated an inhibitory effect on cell proliferation.

**FIGURE 3:**
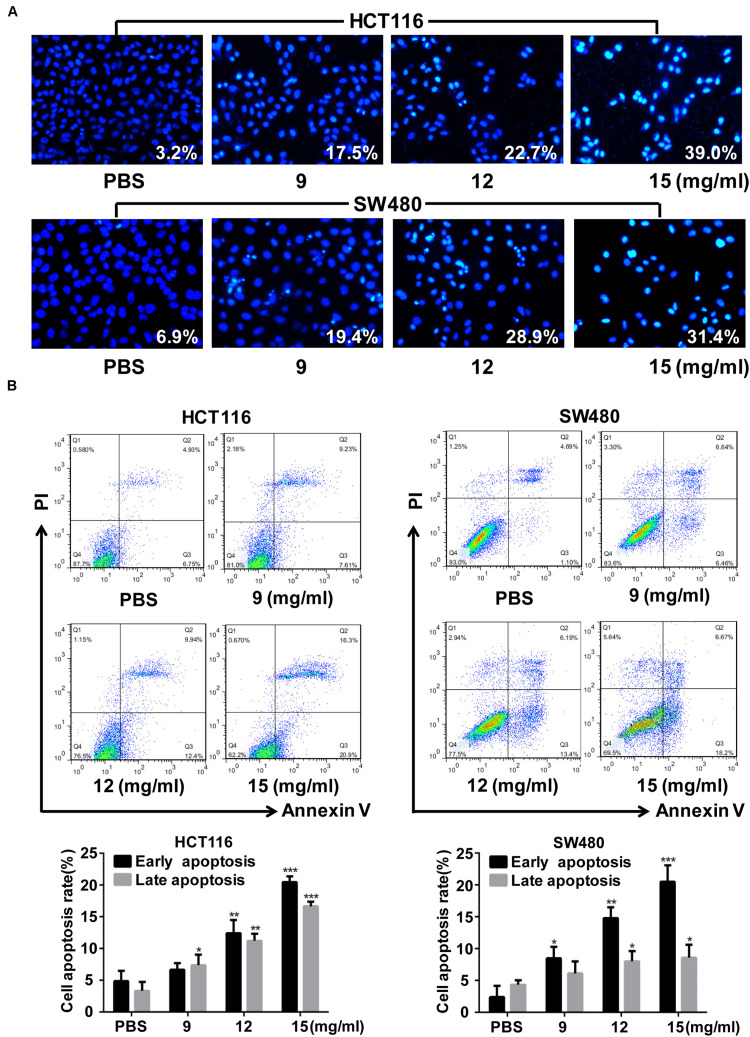
Fufang Yiliu Yin (FYY) promoted colorectal cancer cell apoptosis. **(A)** Hoechst 33258 staining analysis indicated that FYY promoted apoptosis including chromatin condensation and nuclear fragmentation in HCT116 and SW480 cells (×400 magnification). **(B)** Flow cytometry indicated that FYY promoted the early and late apoptosis of HCT116 and SW480 cells (*n* = 3 per group). Values are shown as the mean ± SD, *p < 0.05, **p < 0.01, and ***p < 0.001 vs. control group. The *p*-values were obtained using ANOVA.

Cell apoptosis, as shown by Hoechst staining, increased after FYY treatment (Figure 3A). Flow cytometry analysis showed that the early (*p* = 0.001 for 12 mg/ml) and late apoptosis (*p* = 0.019 for 9 mg/ml) of HCT116 cells were significantly promoted (Figure 3B) by FYY treatment. Similar results were obtained for SW480 cells (Figure 3B).

### Network Pharmacological Analysis of FYY Targeting CRC

**FIGURE 4:**
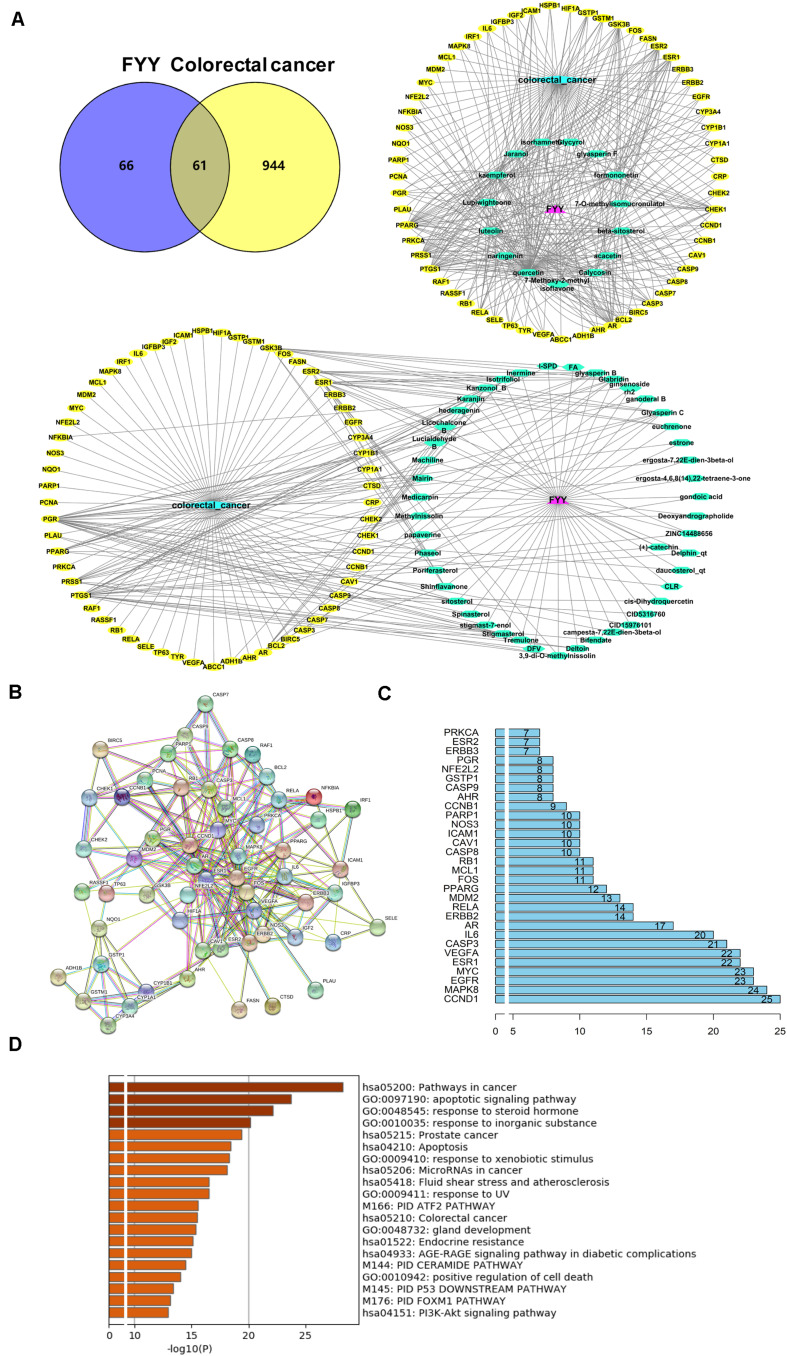
Network pharmacological analysis and biological functional enrichment analysis of Fufang Yiliu Yin (FYY). **(A)** Venn diagram showed 61 common targets of FYY in colorectal cancer (CRC); compound–disease–target networks of FYY against CRC. **(B)** Protein–protein interactions identified by STRING software. **(C)** The predicted key targets of FYY treatment of CRC. **(D)** GO and KEGG pathway enrichment analyses.

A total of 218 compounds from FYY were retrieved (oral bioavailability ≥ 30% and drug likeness ≥ 0.18) from the TCMSP database (Supplementary Table 1). A total of 127 genes related to these compounds, and 1,005 genes related to CRC were screened out. Using Venny 2.1.0 (Figure 4A), 61 common targets were obtained (Supplementary Table 2).

Data imported into Cytoscape 3.5.1 to construct compound–disease–target networks (Figure 4A) showed that 61 of the 218 FYY compounds may affect disease targets. The top 15 core compounds were screened based on the topological properties of degree as shown in Table 1. Quercetin, kaempferol, luteolin, beta-sitosterol, isorhamnetin, formononetin, calycosin, jaranol, acacetin, and naringenin were the top 10 active FYY ingredients against CRC. The other 46 active compounds are listed in Supplementary Table 3. Two networks were constructed for the top 15 core compounds and the remaining 46 active compounds (Figure 4A). The protein–protein interaction network built using STRING software (used to investigate the mechanisms of FYY) provided 61 common targets after setting the confidence level above 0.7 (Figure 4B). The prioritization of key targets was analyzed according to the degree of the node exported from the STRING database, and the top five targets were cyclin-D1, MAPK8, EGFR, MYC, and ESR1 (Figure 4C).

**TABLE 1:** The top 15 bioactive compounds of Fufang Yiliu Yin are listed below according to the degree of similarity of the compound–disease–target networks.

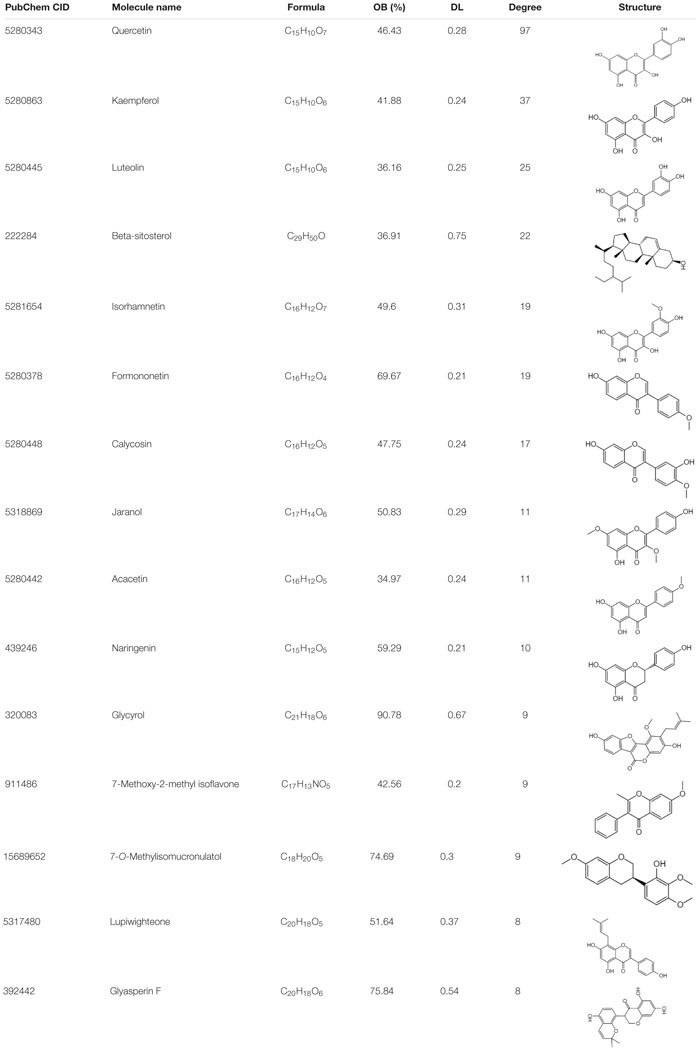

*OB, oral bioavailability; DL, drug-likeness.*

### Biological Function and Pathway Enrichment of FYY on CRC

The biological functions and signaling pathways from all core targets were enriched. The top 20 biological enrichment results are shown in Figure 4D. FYY affected CRC through multiple GO biological processes, including apoptotic signaling pathway, response to steroid hormone, and response to inorganic substance. KEGG analysis results included cancer, prostate cancer, apoptosis, and PI3K/Akt signaling pathways.

**FIGURE 5:**
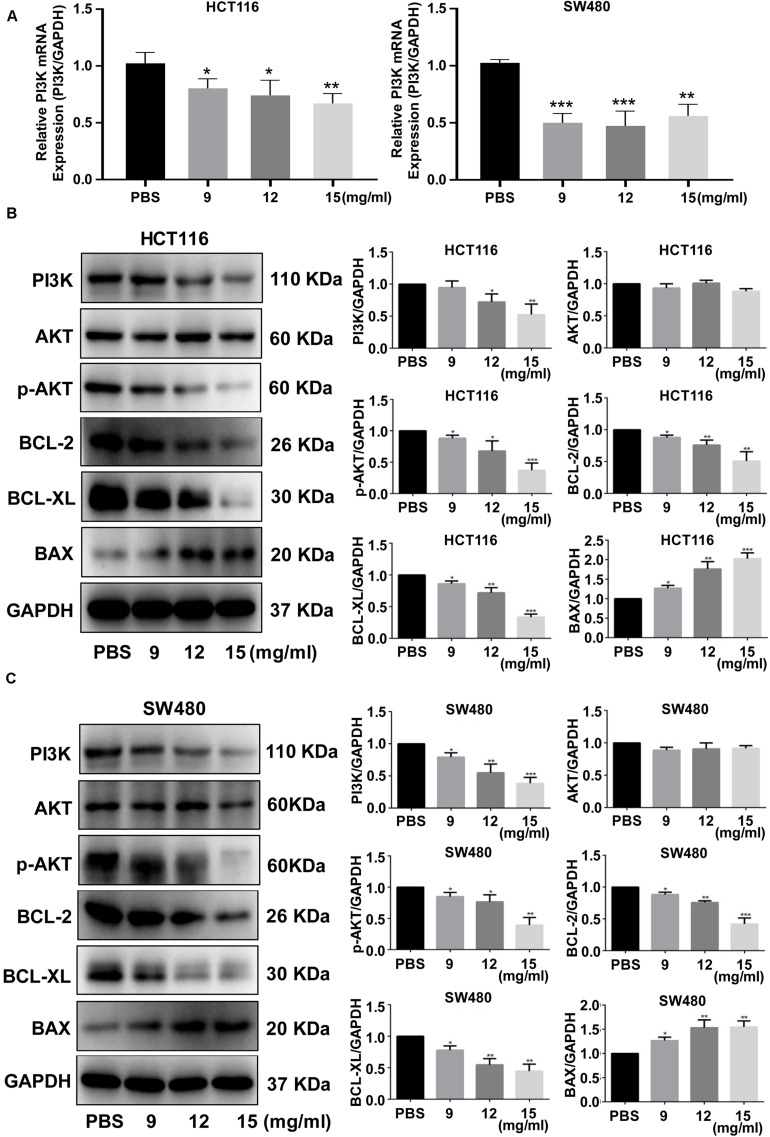
Fufang Yiliu Yin (FYY) modulated the expression of the PI3K/Akt signaling pathway and BCL-2 family proteins. Relative PI3K mRNA expression was altered by FYY treatment in HCT116 and SW480 cells **(A)** (*n* = 3 per group). Expression levels of PI3K, AKT, p-AKT, BCL-2, BCL-XL, and BAX were altered by FYY treatment in HCT116 **(B)** and SW480 cells **(C)** (*n* = 3 per group). Values are shown as the mean ± SD, *p < 0.05, **p < 0.01, and ***p < 0.001 vs. control group. The *p*-values were obtained using ANOVA.

We further investigated how the FYY mechanism promoted apoptosis using RT-PCR and western blot analysis of HCT116 and SW480 cells; FYY inhibited the relative expression of PI3K mRNA (*p* < 0.05, Figure 5A). FYY downregulated the expression of PI3K, p-AKT, BCL-2, and BCL-XL and upregulated the expression of BAX (*p* < 0.05, Figures 5B,C). Taken together, these data support the idea that FYY induces CRC cell apoptosis by modulating the PI3K/Akt pathway and BCL-2 family proteins.

### FYY Inhibited Tumor Growth and Cell Proliferation *in vivo*

**FIGURE 6:**
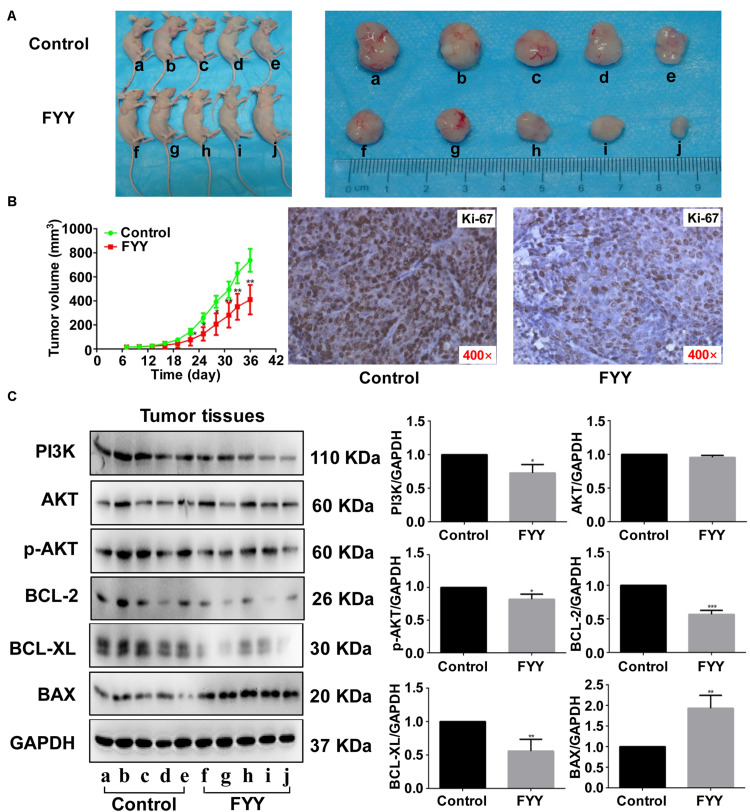
Fufang Yiliu Yin (FYY) inhibited tumor growth *in vivo.* **(A)** Subcutaneous xenograft tumors after 36 days demonstrated that FYY inhibited xenograft tumor growth (*n* = 5 per group). **(B)** Tumor volume was significantly smaller after 14 days of FYY treatment (*n* = 5 per group). IHC analysis of Ki-67 expression in FYY-treated tumor and liver tissues (×400 magnification). The *p*-values were obtained using ANOVA. (C) Protein expression levels of PI3K, AKT, p-AKT, BCL-2, BCL-XL, and BAX in tumor tissues (*n* = 3 per group). Values are shown as the mean ± SD, *p < 0.05 and **p < 0.01 vs. control group. The *p*-values were obtained using Student’s *t*-test.

The HCT116 cell xenograft model used to investigate the antitumor effect of FYY showed that FYY significantly inhibited tumor growth compared to the control (Figure 6A). After 30 days of treatment, the average tumor volumes were 738.00 ± 442.38 mm^3^ in the control group and 411.2.00 ± 54.87 mm^3^ in FYY-treated group (Figure 6B), while tumor weights were 678.00 ± 57.83 and 294.00 ± 73.66 mg, respectively. Ki-67 significantly decreased in the FYY-treated CRC tumor xenograft group (Figure 6B). The expression of PI3K, p-AKT, BCL-2, and BCL-XL followed the same trend as the *in vitro* study results (Figure 6C).

## Discussion

Both retrospective and prospective studies have proven the anticancer effects of TCM on CRC (Shi et al., 2017; Xu et al., 2017). Here, we reported the anticancer effect of the FYY formula, which contains eight ingredients. FYY significantly inhibited cell proliferation and promoted CRC cell apoptosis *in vitro*. FYY also inhibited xenograft tumor growth *in vivo*. Using a network pharmacology analysis, we found that FYY may act on CRC through 61 active compounds targeting 61 CRC-related genes that regulate the apoptosis and PI3K/Akt signaling pathways.

To better understand the complementary effects of FYY formula ingredients, we retrieved a total of 218 compounds from the TCMSP database (Supplementary Table 1). Compound–disease–target networks showed that 61 of the 218 compounds may affect 61 CRC-related targets. By searching PubMed, we found that the top 10 compounds (Table 1) exhibit anti-CRC effects mainly by promoting apoptosis and inhibiting cell proliferation. For example, quercetin was mostly related to protective effects against CRC and is found in three of the eight remedies in FYY, Astragali Radix (Huang Qi), *H. diffusa* Willd (Bai Hua She She Cao), and *G. glabra* Linne (Gan Cao). Quercetin inhibits CRC progression by promoting cell apoptosis and autophagy, as well as inhibiting angiogenesis and inflammation (Darband et al., 2018). Quercetin induces apoptosis by inhibiting different signaling pathways including the MAPK/Erk, PI3K/Akt, and NF-κB signaling pathways (Zhang et al., 2015; Xavier et al., 2019); it also inhibits the migration and invasion of CRC cells via regulating the toll-like receptor 4/NF-κB signaling pathway (Han et al., 2016). Further, kaempferol induces CRC cell apoptosis (Choi et al., 2018), while isorhamnetin, formononetin, and naringenin show anticancer effects by inhibiting cell proliferation (Li et al., 2014; Abaza et al., 2015). The similarity of the effects provided by FYY compounds may provide a mutual enhancement effect, but this must be further tested using single or mixed compounds.

Fufang Yiliu Yin induced cell cycle arrest in CRC cells at the G0/G1 phase and promoted apoptosis in HCT116 and SW480 cells. To explain the mechanism by which FYY inhibits cell proliferation and promotes apoptosis, we performed protein–protein interaction network, KEGG, and GO pathway analyses. Protein–protein interaction network analysis indicated the top five targets were cyclin-D1, MAPK8, EGFR, C-MYC, and ESR1. Biological functional analysis indicated apoptosis and cancer-related pathways including the PI3K/Akt signaling pathway. Then, our experimental study confirmed the activation of the PI3K/Akt pathway and BCL-2 family proteins, as well as C-MYC expression.

**FIGURE 7:**
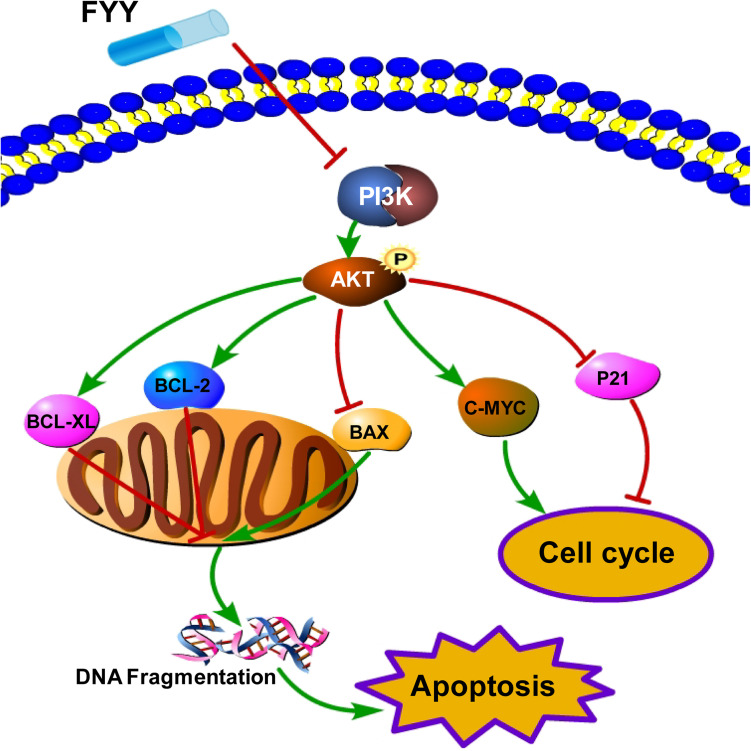
Schematic representation of the proposed PI3K/Akt signaling-induced cell cycle arrest and apoptosis triggered by Fufang Yiliu Yin (FYY). By combining the network pharmacological analysis and our results, we hypothesized that FYY activates the PI3K/Akt signaling pathway and modulates the expression of P21, C-MYC, and BCL-2 family proteins, thereby inducing cell cycle arrest and apoptosis.

Traditional Chinese medicine formulas reportedly inhibit cancer progression by different signaling pathways. A TCM formula, Jianpi Jiedu, inhibits CRC tumorigenesis and metastasis via the mTOR/HIF-1α/VEGF pathway (Peng et al., 2018). Another TCM formula, Huang Qin Ge Gen Tang, enhances the 5-fluorouracil anticancer effect by regulating the E2F1/TS pathway (Liu et al., 2018). The Zhi Zhen Fang formula reverses multidrug resistance mediated by the Hedgehog pathway in CRC (Sui et al., 2017). These formulas, as well as FYY, all contain Astragali Radix (Huang Qi), *H. diffusa* Willd (Bai Hua She She Cao), *G. glabra* Linne (Gan Cao), and Radix Panacis Quinquefolii (Xi Yang Shen). However, there have been no reports regarding the anticancer effect of TCM formulas acting through the apoptosis and PI3K/Akt pathways in CRC (Figure 7). In the current study, we found that FYY decreased the transcription and protein level of PI3K (Figure 5) and further inhibited the phosphorylation of Akt in both the cells and tumor tissues (Figures 5, 6). Accumulating evidence indicates that the PI3K/Akt pathway plays an important role in tumor development. PI3K can partially activate Akt at the Thr308 or Ser473 sites by inducing the translocation of Akt to the cell membrane via phosphoinositide-dependent kinase 1. Akt inhibition is usually indicated by a decrease in the p-Akt (Ser473) level and is mostly achieved by inhibiting PI3K using PI3K-specific inhibitors LY294002 or Wortmannin (Reener and Marti, 2013). The regulation of PI3K/Akt transcription and protein expression by a TCM treatment has been previously reported. TCM intervention decreased p-Akt levels following the concentration gradient of the TCM treatment, while the total overall Akt level was unchanged (Gu et al., 2017; Zhao et al., 2018). Calycosin, a component of Astragali Radix, reportedly inhibits CRC proliferation through the ERβ-mediated regulation of the IGF-1R and PI3K/Akt signaling pathways (Zhao et al., 2016). Quercetin, kaempferol, and rutin in *H. diffusa* Willd also exhibit anticancer effects in CRC by regulating the PI3K/Akt signaling pathway (Cai et al., 2012).

We previously found that FYY inhibited cell proliferation, promoted cell apoptosis, and inhibited metastasis of hepatocellular carcinoma (Yang et al., 2018). FYY may have a similar effect on different types of cancer. Although we demonstrated both the anticancer effects of FYY and the action mechanism by which it operates, limitations of this study include the following: first, we did not investigate the antimetastatic effect of FYY on CRC. A migration and invasion assay and CRC liver metastasis model should be used to investigate this. Second, further studies should investigate whether mutual enhancement effects exist between the applications of FYY and regular chemotherapy and also examine its effect on drug resistance.

In conclusion, our study findings showed that FYY inhibited proliferation and promoted apoptosis in CRC cells by modulating the PI3K/Akt signaling pathway and BCL-2 family proteins. We believe that FYY could be a promising adjuvant therapy for CRC.

## Data Availability Statement

All data presented in this study are included in the article/Supplementary Material.

## Ethics Statement

The animal study was reviewed and approved by Animal Ethics Committee of The Affiliated Hospital of Qingdao University (AHQU20180310A). Written informed consent was obtained from the owners for the participation of their animals in this study.

## Author Contributions

BD and CZ obtained funding, conducted the research, and prepared the manuscript. ZY and QJ performed the experiments. SZ prepared and provided the FYY formula. YW and HZ performed the network pharmacology analysis. CS designed the study and interpreted the data. All authors contributed to the article and approved the submitted version.

## Conflict of Interest

The authors declare that the research was conducted in the absence of any commercial or financial relationships that could be construed as a potential conflict of interest.

## Footnotes

1 http://tcmspw.com/tcmsp.php

2 https://www.genecards.org/

3 https://www.omim.org/

4 http://bioinfogp.cnb.csic.es/tools/venny/
