# Supplementary material for: Anticancer Effects of Fufang Yiliu Yin Formula on Colorectal Cancer Through Modulation of the PI3K/Akt Pathway and BCL-2 Family Proteins
Source: Front Cell Dev Biol. 2020 Aug 11;8:704. doi: 10.3389/fcell.2020.00704 (PMC7431655; doi:10.3389/fcell.2020.00704)
Supplement: Supplementary file 1 [file Table_1.DOCX]

**Supplement Table 1** The 218 compounds of FYY. Compounds were retrieved with the criteria of OB≥30% and DL≥0.18 through TCMSP database.

| Molecule ID | Molecule Name | OB(%) | DL |
| --- | --- | --- | --- |
| MOL001646 | 2,3-dimethoxy-6-methyanthraquinone | 34.86 | 0.26 |
| MOL001659 | Poriferasterol | 43.83 | 0.76 |
| MOL001663 | (4aS,6aR,6aS,6bR,8aR,10R,12aR,14bS)-10-hydroxy-2,2,6a,6b,9,9,12a-heptamethyl-1,3,4,5,6,6a,7,8,8a,10,11,12,13,14b-tetradecahydropicene-4a-carboxylic acid | 32.03 | 0.76 |
| MOL001670 | ZINC14488656 | 37.83 | 0.21 |
| MOL000449 | Stigmasterol | 43.83 | 0.76 |
| MOL000358 | beta-sitosterol | 36.91 | 0.75 |
| MOL000098 | quercetin | 46.43 | 0.28 |
| MOL002211 | 11,14-eicosadienoic acid | 39.99 | 0.2 |
| MOL002388 | Delphin_qt | 57.76 | 0.28 |
| MOL002392 | Deltoin | 46.69 | 0.37 |
| MOL002393 | Demethyldelavaine A | 34.52 | 0.18 |
| MOL002394 | Demethyldelavaine B | 34.52 | 0.18 |
| MOL002395 | Deoxyandrographolide | 56.3 | 0.31 |
| MOL002397 | karakoline | 51.73 | 0.73 |
| MOL002398 | Karanjin | 69.56 | 0.34 |
| MOL002401 | Neokadsuranic acid B | 43.1 | 0.85 |
| MOL002406 | 2,7-Dideacetyl-2,7-dibenzoyl-taxayunnanine F | 39.43 | 0.38 |
| MOL002410 | benzoylnapelline | 34.06 | 0.53 |
| MOL002415 | 6-Demethyldesoline | 51.87 | 0.66 |
| MOL002416 | deoxyaconitine | 30.96 | 0.24 |
| MOL002419 | (R)-Norcoclaurine | 82.54 | 0.21 |
| MOL002421 | ignavine | 84.08 | 0.25 |
| MOL002422 | isotalatizidine | 50.82 | 0.73 |
| MOL002423 | jesaconitine | 33.41 | 0.19 |
| MOL002433 | (3R,8S,9R,10R,13R,14S,17R)-3-hydroxy-4,4,9,13,14-pentamethyl-17-[(E,2R)-6-methyl-7-[(2R,3R,4S,5S,6R)-3,4,5-trihydroxy-6-[[(2R,3R,4S,5S,6R)-3,4,5-trihydroxy-6-(hydroxymethyl)oxan-2-yl]oxymethyl]oxan-2-yl]oxyhept-5-en-2-yl]-1,2,3,7,8,10,12,15,16,17-decahydr | 41.52 | 0.22 |
| MOL002434 | Carnosifloside I_qt | 38.16 | 0.8 |
| MOL000359 | sitosterol | 36.91 | 0.75 |
| MOL000538 | hypaconitine | 31.39 | 0.26 |
| MOL001484 | Inermine | 75.18 | 0.54 |
| MOL001792 | DFV | 32.76 | 0.18 |
| MOL000211 | Mairin | 55.38 | 0.78 |
| MOL002311 | Glycyrol | 90.78 | 0.67 |
| MOL000239 | Jaranol | 50.83 | 0.29 |
| MOL002565 | Medicarpin | 49.22 | 0.34 |
| MOL000354 | isorhamnetin | 49.6 | 0.31 |
| MOL000359 | sitosterol | 36.91 | 0.75 |
| MOL003656 | Lupiwighteone | 51.64 | 0.37 |
| MOL003896 | 7-Methoxy-2-methyl isoflavone | 42.56 | 0.2 |
| MOL000392 | formononetin | 69.67 | 0.21 |
| MOL000417 | Calycosin | 47.75 | 0.24 |
| MOL000422 | kaempferol | 41.88 | 0.24 |
| MOL004328 | naringenin | 59.29 | 0.21 |
| MOL004805 | Shinflavanone | 31.79 | 0.72 |
| MOL004806 | euchrenone | 30.29 | 0.57 |
| MOL004808 | glyasperin B | 65.22 | 0.44 |
| MOL004810 | glyasperin F | 75.84 | 0.54 |
| MOL004811 | Glyasperin C | 45.56 | 0.4 |
| MOL004814 | Isotrifoliol | 31.94 | 0.42 |
| MOL004815 | Kanzonol B | 39.62 | 0.35 |
| MOL004820 | kanzonols W | 50.48 | 0.52 |
| MOL004824 | (2S)-6-(2,4-dihydroxyphenyl)-2-(2-hydroxypropan-2-yl)-4-methoxy-2,3-dihydrofuro[3,2-g]chromen-7-one | 60.25 | 0.63 |
| MOL004827 | Semilicoisoflavone B | 48.78 | 0.55 |
| MOL004828 | Glepidotin A | 44.72 | 0.35 |
| MOL004829 | Glepidotin B | 64.46 | 0.34 |
| MOL004833 | Phaseolinisoflavan | 32.01 | 0.45 |
| MOL004835 | Glypallichalcone | 61.6 | 0.19 |
| MOL004838 | 8-(6-hydroxy-2-benzofuranyl)-2,2-dimethyl-5-chromenol | 58.44 | 0.38 |
| MOL004841 | Licochalcone B | 76.76 | 0.19 |
| MOL004848 | licochalcone G | 49.25 | 0.32 |
| MOL004849 | 3-(2,4-dihydroxyphenyl)-8-(1,1-dimethylprop-2-enyl)-7-hydroxy-5-methoxy-coumarin | 59.62 | 0.43 |
| MOL004855 | Licoricone | 63.58 | 0.47 |
| MOL004856 | Gancaonin A | 51.08 | 0.4 |
| MOL004857 | Gancaonin B | 48.79 | 0.45 |
| MOL004860 | licorice glycoside E | 32.89 | 0.27 |
| MOL004863 | 3-(3,4-dihydroxyphenyl)-5,7-dihydroxy-8-(3-methylbut-2-enyl)chromone | 66.37 | 0.41 |
| MOL004864 | 5,7-dihydroxy-3-(4-methoxyphenyl)-8-(3-methylbut-2-enyl)chromone | 30.49 | 0.41 |
| MOL004866 | 2-(3,4-dihydroxyphenyl)-5,7-dihydroxy-6-(3-methylbut-2-enyl)chromone | 44.15 | 0.41 |
| MOL004879 | Glycyrin | 52.61 | 0.47 |
| MOL004882 | Licocoumarone | 33.21 | 0.36 |
| MOL004883 | Licoisoflavone | 41.61 | 0.42 |
| MOL004884 | Licoisoflavone B | 38.93 | 0.55 |
| MOL004885 | licoisoflavanone | 52.47 | 0.54 |
| MOL004891 | shinpterocarpin | 80.3 | 0.73 |
| MOL004898 | (E)-3-[3,4-dihydroxy-5-(3-methylbut-2-enyl)phenyl]-1-(2,4-dihydroxyphenyl)prop-2-en-1-one | 46.27 | 0.31 |
| MOL004903 | liquiritin | 65.69 | 0.74 |
| MOL004904 | licopyranocoumarin | 80.36 | 0.65 |
| MOL004905 | 3,22-Dihydroxy-11-oxo-delta(12)-oleanene-27-alpha-methoxycarbonyl-29-oic acid | 34.32 | 0.55 |
| MOL004907 | Glyzaglabrin | 61.07 | 0.35 |
| MOL004908 | Glabridin | 53.25 | 0.47 |
| MOL004910 | Glabranin | 52.9 | 0.31 |
| MOL004911 | Glabrene | 46.27 | 0.44 |
| MOL004912 | Glabrone | 52.51 | 0.5 |
| MOL004913 | 1,3-dihydroxy-9-methoxy-6-benzofurano[3,2-c]chromenone | 48.14 | 0.43 |
| MOL004914 | 1,3-dihydroxy-8,9-dimethoxy-6-benzofurano[3,2-c]chromenone | 62.9 | 0.53 |
| MOL004915 | Eurycarpin A | 43.28 | 0.37 |
| MOL004917 | glycyroside | 37.25 | 0.79 |
| MOL004924 | (-)-Medicocarpin | 40.99 | 0.95 |
| MOL004935 | Sigmoidin-B | 34.88 | 0.41 |
| MOL004941 | (2R)-7-hydroxy-2-(4-hydroxyphenyl)chroman-4-one | 71.12 | 0.18 |
| MOL004945 | (2S)-7-hydroxy-2-(4-hydroxyphenyl)-8-(3-methylbut-2-enyl)chroman-4-one | 36.57 | 0.32 |
| MOL004948 | Isoglycyrol | 44.7 | 0.84 |
| MOL004949 | Isolicoflavonol | 45.17 | 0.42 |
| MOL004957 | HMO | 38.37 | 0.21 |
| MOL004959 | 1-Methoxyphaseollidin | 69.98 | 0.64 |
| MOL004961 | Quercetin der. | 46.45 | 0.33 |
| MOL004966 | 3'-Hydroxy-4'-O-Methylglabridin | 43.71 | 0.57 |
| MOL000497 | licochalcone a | 40.79 | 0.29 |
| MOL004974 | 3'-Methoxyglabridin | 46.16 | 0.57 |
| MOL004978 | 2-[(3R)-8,8-dimethyl-3,4-dihydro-2H-pyrano[6,5-f]chromen-3-yl]-5-methoxyphenol | 36.21 | 0.52 |
| MOL004980 | Inflacoumarin A | 39.71 | 0.33 |
| MOL004985 | icos-5-enoic acid | 30.7 | 0.2 |
| MOL004988 | Kanzonol F | 32.47 | 0.89 |
| MOL004989 | 6-prenylated eriodictyol | 39.22 | 0.41 |
| MOL004990 | 7,2',4'-trihydroxy－5-methoxy-3－arylcoumarin | 83.71 | 0.27 |
| MOL004991 | 7-Acetoxy-2-methylisoflavone | 38.92 | 0.26 |
| MOL004993 | 8-prenylated eriodictyol | 53.79 | 0.4 |
| MOL004996 | gadelaidic acid | 30.7 | 0.2 |
| MOL000500 | Vestitol | 74.66 | 0.21 |
| MOL005000 | Gancaonin G | 60.44 | 0.39 |
| MOL005001 | Gancaonin H | 50.1 | 0.78 |
| MOL005003 | Licoagrocarpin | 58.81 | 0.58 |
| MOL005007 | Glyasperins M | 72.67 | 0.59 |
| MOL005008 | Glycyrrhiza flavonol A | 41.28 | 0.6 |
| MOL005012 | Licoagroisoflavone | 57.28 | 0.49 |
| MOL005013 | 18α-hydroxyglycyrrhetic acid | 41.16 | 0.71 |
| MOL005016 | Odoratin | 49.95 | 0.3 |
| MOL005017 | Phaseol | 78.77 | 0.58 |
| MOL005018 | Xambioona | 54.85 | 0.87 |
| MOL005020 | dehydroglyasperins C | 53.82 | 0.37 |
| MOL000098 | quercetin | 46.43 | 0.28 |
| MOL000211 | Mairin | 55.38 | 0.78 |
| MOL000239 | Jaranol | 50.83 | 0.29 |
| MOL000296 | hederagenin | 36.91 | 0.75 |
| MOL000033 | CID15976101 | 36.23 | 0.78 |
| MOL000354 | isorhamnetin | 49.6 | 0.31 |
| MOL000371 | 3,9-di-O-methylnissolin | 53.74 | 0.48 |
| MOL000374 | 5'-hydroxyiso-muronulatol-2',5'-di-O-glucoside | 41.72 | 0.69 |
| MOL000378 | 7-O-methylisomucronulatol | 74.69 | 0.3 |
| MOL000379 | 9,10-dimethoxypterocarpan-3-O-β-D-glucoside | 36.74 | 0.92 |
| MOL000380 | Methylnissolin | 64.26 | 0.42 |
| MOL000387 | Bifendate | 31.1 | 0.67 |
| MOL000392 | formononetin | 69.67 | 0.21 |
| MOL000398 | isoflavanone | 109.99 | 0.3 |
| MOL000417 | Calycosin | 47.75 | 0.24 |
| MOL000422 | kaempferol | 41.88 | 0.24 |
| MOL000433 | FA | 68.96 | 0.71 |
| MOL000438 | (3R)-3-(2-hydroxy-3,4-dimethoxyphenyl)chroman-7-ol | 67.67 | 0.26 |
| MOL000439 | isomucronulatol-7,2'-di-O-glucosiole | 49.28 | 0.62 |
| MOL000442 | CID5316760 | 39.05 | 0.48 |
| MOL000098 | quercetin | 46.43 | 0.28 |
| MOL001689 | acacetin | 34.97 | 0.24 |
| MOL004355 | Spinasterol | 42.98 | 0.76 |
| MOL004580 | cis-Dihydroquercetin | 66.44 | 0.27 |
| MOL005996 | 2-O-methyl-3―O-β-D-glucopyranosyl platycogenate A | 45.15 | 0.25 |
| MOL000006 | luteolin | 36.16 | 0.25 |
| MOL006026 | dimethyl 2-O-methyl-3-O-a-D-glucopyranosyl platycogenate A | 39.21 | 0.25 |
| MOL006070 | robinin | 39.84 | 0.71 |
| MOL010921 | estrone | 53.56 | 0.32 |
| MOL010922 | Diisooctyl succinate | 31.62 | 0.23 |
| MOL002211 | 11,14-eicosadienoic acid | 39.99 | 0.2 |
| MOL002372 | (6Z,10E,14E,18E)-2,6,10,15,19,23-hexamethyltetracosa-2,6,10,14,18,22-hexaene | 33.55 | 0.42 |
| MOL000359 | sitosterol | 36.91 | 0.75 |
| MOL000449 | Stigmasterol | 43.83 | 0.76 |
| MOL005030 | gondoic acid | 30.7 | 0.2 |
| MOL000953 | CLR | 37.87 | 0.68 |
| MOL000211 | Mairin | 55.38 | 0.78 |
| MOL000492 | (+)-catechin | 54.83 | 0.24 |
| MOL002311 | Glycyrol | 90.78 | 0.67 |
| MOL003410 | Ziziphin_qt | 66.95 | 0.62 |
| MOL004355 | Spinasterol | 42.98 | 0.76 |
| MOL004841 | Licochalcone B | 76.76 | 0.19 |
| MOL004903 | liquiritin | 65.69 | 0.74 |
| MOL004908 | Glabridin | 53.25 | 0.47 |
| MOL005017 | Phaseol | 78.77 | 0.58 |
| MOL007207 | Machiline | 79.64 | 0.24 |
| MOL012922 | l-SPD | 87.35 | 0.54 |
| MOL011125 | (+)-Ganoderic acid Mf | 32.62 | 0.82 |
| MOL011127 | (+)-Methyl ganolucidate A | 31.14 | 0.82 |
| MOL011129 | methyl (4R)-4-[(5R,10S,13R,14R,17R)-4,4,10,13,14-pentamethyl-3,7,11,15-tetraoxo-2,5,6,12,16,17-hexahydro-1H-cyclopenta[a]phenanthren-17-yl]pentanoate | 32.67 | 0.81 |
| MOL011135 | 22,23-dimethylene ganodermic acid S | 33.61 | 0.71 |
| MOL011136 | 22β-acetoxy-3α,15α-dihydroxylanosta-7,9(11),24-trien-26-oic acid | 37.64 | 0.8 |
| MOL011137 | campesta-7,22E-dien-3beta-ol | 43.51 | 0.72 |
| MOL011140 | 5alpha-Lanosta-7,9(11),24-triene-15alpha,26-dihydroxy-3-one | 38.54 | 0.81 |
| MOL011156 | epoxyganoderiol A | 33.78 | 0.83 |
| MOL011157 | epoxyganoderiol B | 42.3 | 0.83 |
| MOL011158 | epoxyganoderiol C | 37.7 | 0.83 |
| MOL011159 | ergosta-4,6,8(14),22-tetraene-3-one | 48.32 | 0.75 |
| MOL011160 | ergosta-4,7,22-trien-3,6-dione | 47.86 | 0.77 |
| MOL011162 | Ergosta-7,22-dien-3beta-yl palmitate | 37.6 | 0.43 |
| MOL011164 | ergosta-7,22-dien-3β,5α,6α-triol | 31.43 | 0.77 |
| MOL011165 | ergosta-7,22-diene-3beta-yl linoleate | 45.11 | 0.37 |
| MOL011166 | ergosta-7,22-diene-3beta-yl palmitate | 37.6 | 0.43 |
| MOL011167 | ergosta-7,22-diene-3beta-yl pentadecanoate | 38.25 | 0.47 |
| MOL011168 | ergosta-7,9(11),22-trien-3β,5α,6α-triol | 46.95 | 0.78 |
| MOL011169 | Peroxyergosterol | 44.39 | 0.82 |
| MOL011171 | ganoderal B | 42.56 | 0.81 |
| MOL011172 | ganoderan B | 42.19 | 0.81 |
| MOL011183 | (E,6R)-6-[(3S,5R,7S,10S,13R,14R,17R)-3,7-dihydroxy-4,4,10,13,14-pentamethyl-11,15-dioxo-2,3,5,6,7,12,16,17-octahydro-1H-cyclopenta[a]phenanthren-17-yl]-2-methylhept-2-enoic acid | 30.54 | 0.82 |
| MOL011189 | Ganoderic acid DM | 38.8 | 0.83 |
| MOL011206 | (E,6R)-6-[(3R,5R,10S,13R,14R,15S,17R)-3,15-diacetoxy-4,4,10,13,14-pentamethyl-2,3,5,6,12,15,16,17-octahydro-1H-cyclopenta[a]phenanthren-17-yl]-2-methylhept-2-enoic acid | 31.16 | 0.76 |
| MOL011209 | ganoderic acid Mi | 34.4 | 0.81 |
| MOL011214 | (E,5S,6S)-5-acetoxy-6-[(3R,5R,10S,13R,14R,17R)-3-acetoxy-4,4,10,13,14-pentamethyl-2,3,5,6,12,15,16,17-octahydro-1H-cyclopenta[a]phenanthren-17-yl]-2-methylhept-2-enoic acid | 31.46 | 0.77 |
| MOL011215 | (E,5S,6S)-5-acetoxy-6-[(3R,5R,10S,13R,14R,17R)-3-hydroxy-4,4,10,13,14-pentamethyl-2,3,5,6,12,15,16,17-octahydro-1H-cyclopenta[a]phenanthren-17-yl]-2-methylhept-2-enoic acid | 34.37 | 0.83 |
| MOL011218 | ganoderic acid TQ | 36.3 | 0.78 |
| MOL011219 | Ganoderic acid TR | 36.23 | 0.83 |
| MOL011221 | ganoderic acid V | 30.19 | 0.8 |
| MOL011222 | Ganoderic acid V1 | 30.18 | 0.8 |
| MOL011224 | Ganoderic acid X | 33.55 | 0.81 |
| MOL011225 | Ganoderic acid Y | 38.64 | 0.82 |
| MOL011226 | ganoderic acid Z | 37.67 | 0.82 |
| MOL011229 | Ganoderic aldehyde A | 42.26 | 0.81 |
| MOL011235 | Ganoderiol F | 38.12 | 0.82 |
| MOL011241 | Ganodermanondiol | 37.64 | 0.8 |
| MOL011243 | ganodermatriol | 30.46 | 0.82 |
| MOL011244 | ganodermenonol | 44.69 | 0.8 |
| MOL011245 | Ganodermic acid R | 31.16 | 0.76 |
| MOL011247 | ganodermic acid T- Q | 33.55 | 0.81 |
| MOL011248 | ganodermic acid T- O | 32.62 | 0.82 |
| MOL011250 | Ganodermnonol | 44.69 | 0.8 |
| MOL011251 | (5R,10S,13R,14R,17R)-17-[(E,2R)-7-hydroxy-6-methylhept-5-en-2-yl]-4,4,10,13,14-pentamethyl-1,2,5,6,12,15,16,17-octahydrocyclopenta[a]phenanthren-3-one | 44.69 | 0.8 |
| MOL011256 | ganolucidic acid E | 32.85 | 0.82 |
| MOL011258 | ganosporelactone B | 31.21 | 0.33 |
| MOL011262 | lanosta-7,9(11),24-trien-15α-acetoxy-3α-hydroxy-23-oxo-26-oic acid | 31.86 | 0.8 |
| MOL011264 | lanosta-7,9(11),24-trien-3α-acetoxy-15α-hydroxy-23-oxo-26-oic acid | 30.81 | 0.81 |
| MOL011265 | lanosta-7,9(11),24-trien-3α-acetoxy-26-oic acid | 38.85 | 0.84 |
| MOL011266 | Lucialdehyde A | 44.78 | 0.8 |
| MOL011267 | Lucialdehyde B | 43.12 | 0.81 |
| MOL011268 | Lucialdehyde C | 42.26 | 0.81 |
| MOL011270 | (4R)-4-[(5R,7S,10S,13R,14R,17R)-7-hydroxy-3,11,15-triketo-4,4,10,13,14-pentamethyl-1,2,5,6,7,12,16,17-octahydrocyclopenta[a]phenanthren-17-yl]valeric acid | 30.34 | 0.79 |
| MOL011287 | lucidone A | 37.22 | 0.64 |
| MOL011290 | Lucidumol A | 34.75 | 0.8 |
| MOL011303 | methyl Ganoderic acid DM | 39.55 | 0.83 |
| MOL011304 | methyl Ganoderic acid TR | 39.82 | 0.83 |
| MOL011309 | methyl (4R)-4-[(5R,7S,10S,13R,14R,15S,17R)-7,15-dihydroxy-4,4,10,13,14-pentamethyl-3,11-dioxo-2,5,6,7,12,15,16,17-octahydro-1H-cyclopenta[a]phenanthren-17-yl]pentanoate | 30.19 | 0.81 |
| MOL000279 | Cerevisterol | 37.96 | 0.77 |
| MOL000282 | ergosta-7,22E-dien-3beta-ol | 43.51 | 0.72 |
| MOL000358 | beta-sitosterol | 36.91 | 0.75 |
| MOL011394 | (2R,3S,4S,5R,6R)-2-(hydroxymethyl)-6-[[(3S,5R,8R,9R,10R,12R,13R,14R,17S)-12-hydroxy-4,4,8,10,14-pentamethyl-17-[(2S)-6-methyl-2-[(2S,3R,4S,5S,6R)-3,4,5-trihydroxy-6-(hydroxymethyl)oxan-2-yl]oxyhept-5-en-2-yl]-2,3,5,6,7,9,11,12,13,15,16,17-dodecahydro-1H-c | 36.43 | 0.25 |
| MOL011434 | polyacetylene PQ-2 | 36.74 | 0.2 |
| MOL011435 | PQ-2 | 36.74 | 0.19 |
| MOL011442 | Tremulone | 43.87 | 0.75 |
| MOL011455 | 20-Hexadecanoylingenol | 32.7 | 0.65 |
| MOL000358 | beta-sitosterol | 36.91 | 0.75 |
| MOL005344 | ginsenoside rh2 | 36.32 | 0.56 |
| MOL006774 | stigmast-7-enol | 37.42 | 0.75 |
| MOL006980 | papaverine | 64.04 | 0.38 |
| MOL008173 | daucosterol_qt | 36.91 | 0.75 |
| MOL008397 | Daturilin | 50.37 | 0.77 |
