# Supplementary material for: Anticancer Effects of Fufang Yiliu Yin Formula on Colorectal Cancer Through Modulation of the PI3K/Akt Pathway and BCL-2 Family Proteins
Source: Front Cell Dev Biol. 2020 Aug 11;8:704. doi: 10.3389/fcell.2020.00704 (PMC7431655; doi:10.3389/fcell.2020.00704)
Supplement: Supplementary file 2 [file Table_2.DOCX]

**Supplement Table 2** The 61 common targets of FYY and CRC.

| Gene ID | Symbol | Name |
| --- | --- | --- |
| 5241 | PGR | Progesterone receptor |
| 5742 | PTGS1 | Prostaglandin G/H synthase 1 |
| 2099 | ESR1 | Estrogen receptor |
| 5328 | PLAU | Urokinase-type plasminogen activator |
| 596 | BCL2 | Apoptosis regulator Bcl-2 |
| 842 | CASP9 | Caspase-9 |
| 836 | CASP3 | Caspase-3 |
| 841 | CASP8 | Caspase-8 |
| 5578 | PRKCA | Protein kinase C alpha type |
| 367 | AR | Androgen receptor |
| 5468 | PPARG | Peroxisome proliferator activated receptor gamma |
| 5644 | PRSS1 | Trypsin-1 |
| 5970 | RELA | Transcription factor p65 |
| 1956 | EGFR | Epidermal growth factor receptor |
| 7422 | VEGFA | Vascular endothelial growth factor A |
| 595 | CCND1 | G1/S-specific cyclin-D1 |
| 2353 | FOS | Proto-oncogene c-Fos |
| 5925 | RB1 | Retinoblastoma-associated protein |
| 3569 | IL6 | Interleukin-6 |
| 8626 | TP63 | Cellular tumor antigen p53 |
| 4792 | NFKBIA | NF-kappa-B inhibitor alpha |
| 5894 | RAF1 | RAF proto-oncogene serine/threonine-protein kinase |
| 3091 | HIF1A | Hypoxia-inducible factor 1-alpha |
| 2064 | ERBB2 | Receptor tyrosine-protein kinase erbB-2 |
| 1576 | CYP3A4 | Cytochrome P450 3A4 |
| 857 | CAV1 | Caveolin-1 |
| 4609 | MYC | Myc proto-oncogene protein |
| 1543 | CYP1A1 | Cytochrome P450 1A1 |
| 3383 | ICAM1 | Intercellular adhesion molecule 1 |
| 6401 | SELE | E-selectin |
| 332 | BIRC5 | Baculoviral IAP repeat-containing protein 5 |
| 4846 | NOS3 | Nitric oxide synthase, endothelial |
| 3315 | HSPB1 | Heat shock protein beta-1 |
| 1545 | CYP1B1 | Cytochrome P450 1B1 |
| 891 | CCNB1 | G2/mitotic-specific cyclin-B1 |
| 2950 | GSTP1 | Glutathione S-transferase P |
| 4780 | NFE2L2 | Nuclear factor erythroid 2-related factor 2 |
| 1728 | NQO1 | NAD(P)H dehydrogenase [quinone] 1 |
| 142 | PARP1 | Poly [ADP-ribose] polymerase 1 |
| 196 | AHR | Aryl hydrocarbon receptor |
| 11200 | CHEK2 | Serine/threonine-protein kinase Chk2 |
| 1401 | CRP | C-reactive protein |
| 11186 | RASSF1 | Ras association domain-containing protein 1 |
| 1509 | CTSD | Cathepsin D |
| 3486 | IGFBP3 | Insulin-like growth factor-binding protein 3 |
| 3481 | IGF2 | Insulin-like growth factor II |
| 3659 | IRF1 | Interferon regulatory factor 1 |
| 2065 | ERBB3 | Receptor tyrosine-protein kinase erbB-3 |
| 2944 | GSTM1 | Glutathione S-transferase Mu 1 |
| 1111 | CHEK1 | Serine/threonine-protein kinase Chk1 |
| 2932 | GSK3B | Glycogen synthase kinase-3 beta |
| 2100 | ESR2 | Estrogen receptor beta |
| 5599 | MAPK8 | Mitogen-activated protein kinase 8 |
| 2194 | FASN | Fatty acid synthase |
| 4363 | ABCC1 | Multidrug resistance-associated protein 1 |
| 125 | ADH1B | Alcohol dehydrogenase 1B |
| 4193 | MDM2 | E3 ubiquitin-protein ligase Mdm2 |
| 5111 | PCNA | Proliferating cell nuclear antigen |
| 840 | CASP7 | Caspase-7 |
| 4170 | MCL1 | Induced myeloid leukemia cell differentiation protein Mcl-1 |
| 7299 | TYR | Tyrosinase |
