# Supplementary material for: Anticancer Effects of Fufang Yiliu Yin Formula on Colorectal Cancer Through Modulation of the PI3K/Akt Pathway and BCL-2 Family Proteins
Source: Front Cell Dev Biol. 2020 Aug 11;8:704. doi: 10.3389/fcell.2020.00704 (PMC7431655; doi:10.3389/fcell.2020.00704)
Supplement: Supplementary file 3 [file Table_3.DOCX]

**Supplement Table 3** The rest 46 active compounds of FYY. According to the degrees of the compound-disease-target network, the rest 46 active compounds of FYY were listed.

| PubChem CID | Molecule name | Formula | Oral bioavailability (%) | Drug likeness | Degree | Structure |
| --- | --- | --- | --- | --- | --- | --- |
| 480859 | Glyasperin C | C21H24O5 | 45.56 | 0.4 | 8 | 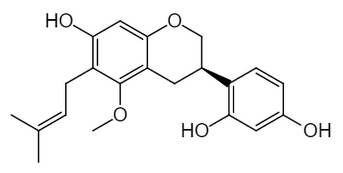 |
| 10881804 | Kanzonol B | C20H18O4 | 39.62 | 0.35 | 8 | 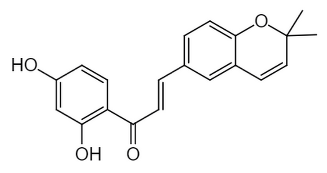 |
| 5318999 | Licochalcone B | C16H14O5 | 76.76 | 0.19 | 8 | 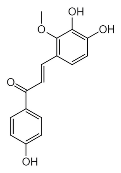 |
| 124052 | Glabridin | C20H20O4 | 53.25 | 0.47 | 8 | 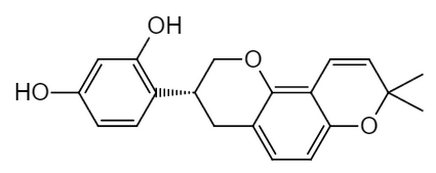 |
| 5280794 | Stigmasterol | C29H48O | 43.83 | 0.76 | 7 | 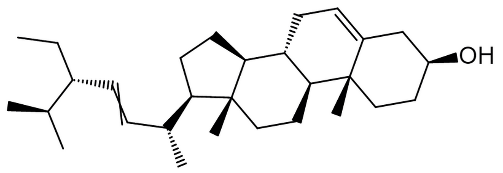 |
| 480784 | glyasperin B | C21H22O6 | 65.22 | 0.44 | 7 | 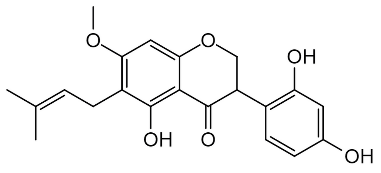 |
| [197678](https://pubchem.ncbi.nlm.nih.gov/compound/197678) | Shinflavanone | [C25H26O4](https://pubchem.ncbi.nlm.nih.gov/search/#query=C25H26O4) | 31.79 | 0.72 | 6 | 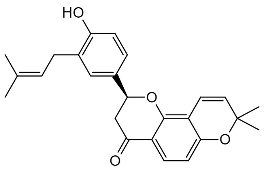 |
| [5318679](https://pubchem.ncbi.nlm.nih.gov/compound/5318679) | Isotrifoliol | [C16H10O6](https://pubchem.ncbi.nlm.nih.gov/search/#query=C16H10O6) | 31.94 | 0.42 | 6 | 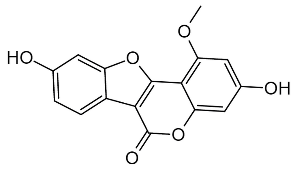 |
| [44257530](https://pubchem.ncbi.nlm.nih.gov/compound/44257530) | Phaseol | [C20H16O5](https://pubchem.ncbi.nlm.nih.gov/search/#query=C20H16O5) | 78.77 | 0.58 | 6 | 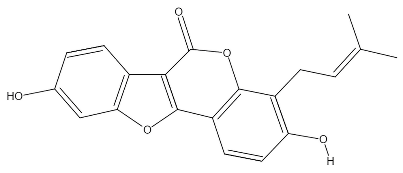 |
| [336327](https://pubchem.ncbi.nlm.nih.gov/compound/336327) | Medicarpin | [C16H14O4](https://pubchem.ncbi.nlm.nih.gov/search/#query=C16H14O4) | 49.22 | 0.34 | 5 | 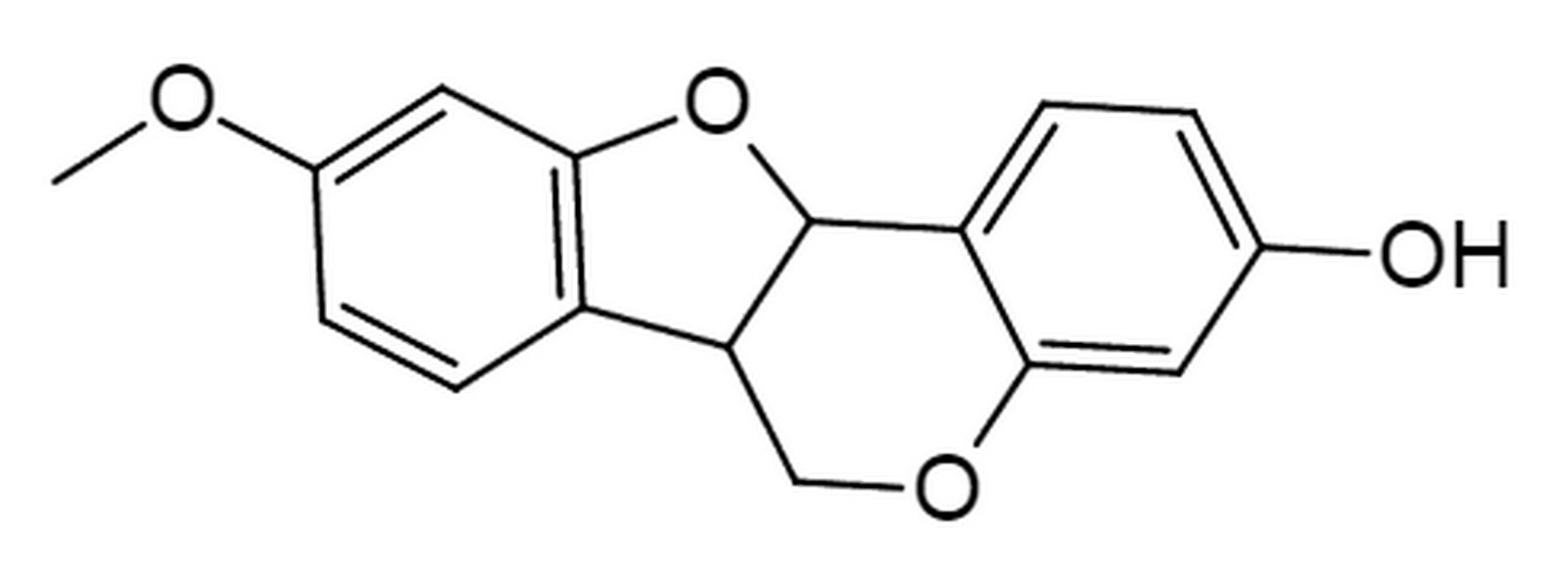 |
| [100633](https://pubchem.ncbi.nlm.nih.gov/compound/100633) | Karanjin | [C18H12O4](https://pubchem.ncbi.nlm.nih.gov/search/#query=C18H12O4) | 69.56 | 0.34 | 4 | 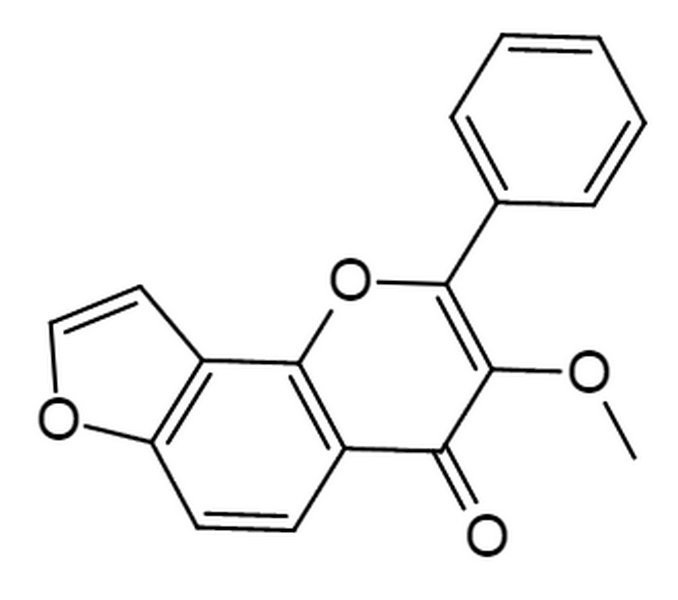 |
| [222284](https://pubchem.ncbi.nlm.nih.gov/compound/222284) | sitosterol | [C29H50O](https://pubchem.ncbi.nlm.nih.gov/search/#query=C29H50O) | 36.91 | 0.75 | 4 | 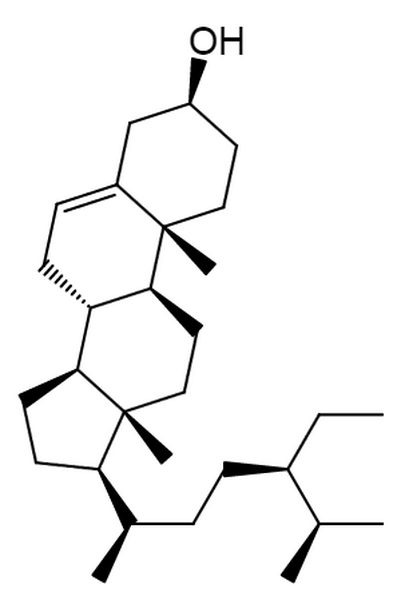 |
| [64971](https://pubchem.ncbi.nlm.nih.gov/compound/64971) | Mairin | [C30H48O3](https://pubchem.ncbi.nlm.nih.gov/search/#query=C30H48O3) | 55.38 | 0.78 | 4 |  |
| [73299](https://pubchem.ncbi.nlm.nih.gov/compound/73299) | hederagenin | [C30H48O4](https://pubchem.ncbi.nlm.nih.gov/search/#query=C30H48O4) | 36.91 | 0.75 | 4 |  |
| [15689655](https://pubchem.ncbi.nlm.nih.gov/compound/15689655) | 3,9-di-O-methylnissolin | [C18H18O5](https://pubchem.ncbi.nlm.nih.gov/search/#query=C18H18O5) | 53.74 | 0.48 | 4 | 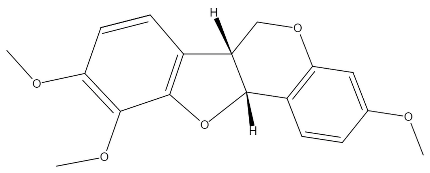 |
| 5319733 | Methylnissolin | C17H16O5 | 64.26 | 0.42 | 4 |  |
| 10514946 | ZINC14488656 | C16H12O3 | 37.83 | 0.21 | 3 | 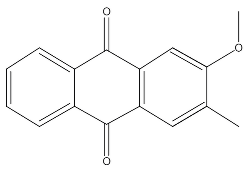 |
| [906525](https://pubchem.ncbi.nlm.nih.gov/compound/906525) | Deltoin | [C19H20O5](https://pubchem.ncbi.nlm.nih.gov/search/#query=C19H20O5) | 46.69 | 0.37 | 3 | 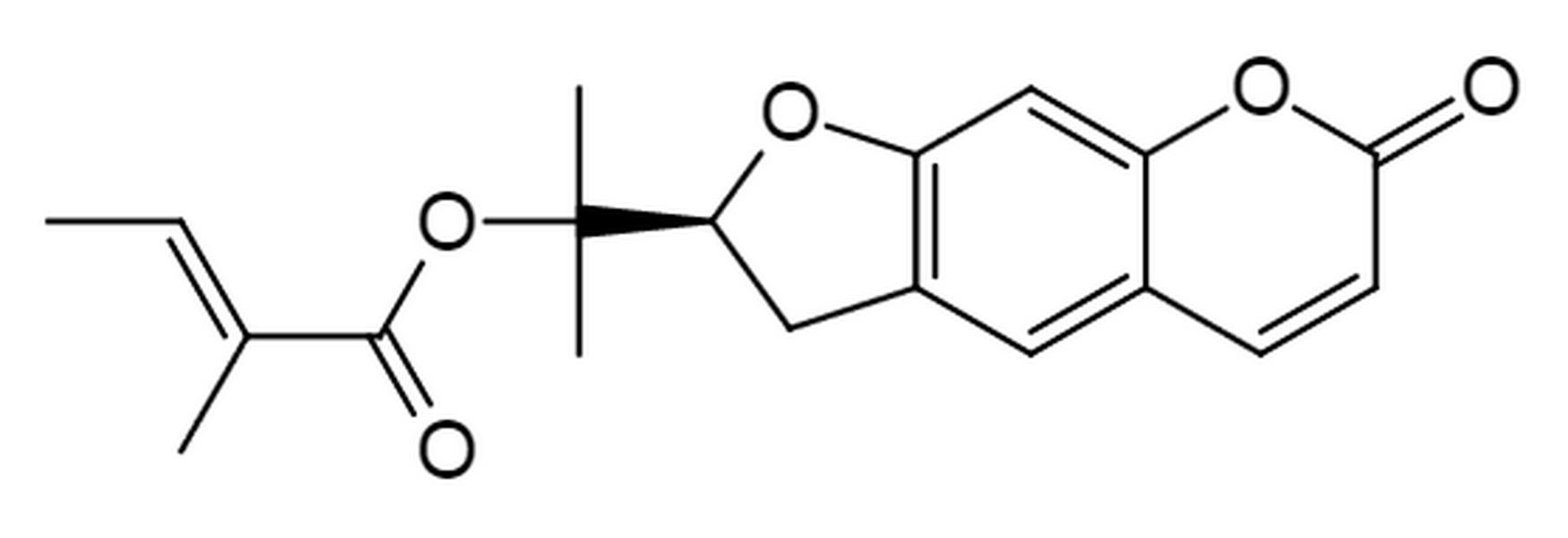 |
| [91510](https://pubchem.ncbi.nlm.nih.gov/compound/91510) | Inermine | [C16H12O5](https://pubchem.ncbi.nlm.nih.gov/search/#query=C16H12O5) | 75.18 | 0.54 | 3 | 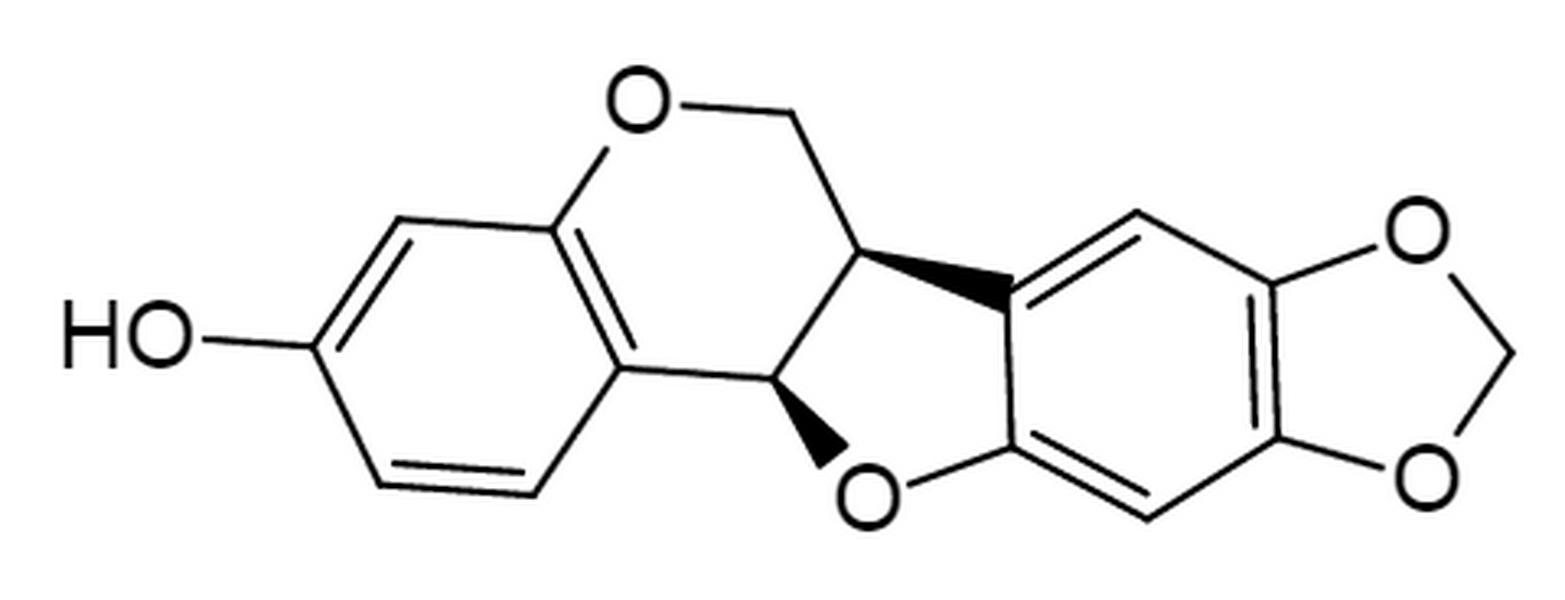 |
| 114829 | Liquiritigenin | C15H12O4 | 32.76 | 0.18 | 3 | 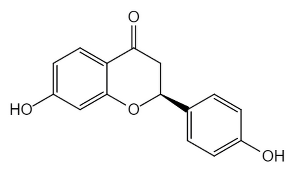 |
| 636551 | Euchrenone A16 | C25H28O5 | 30.29 | 0.57 | 3 | 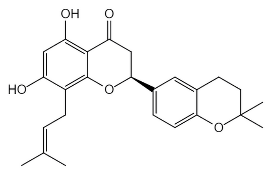 |
| [5281331](https://pubchem.ncbi.nlm.nih.gov/compound/5281331) | Spinasterol | [C29H48O](https://pubchem.ncbi.nlm.nih.gov/search/#query=C29H48O) | 42.98 | 0.76 | 3 | 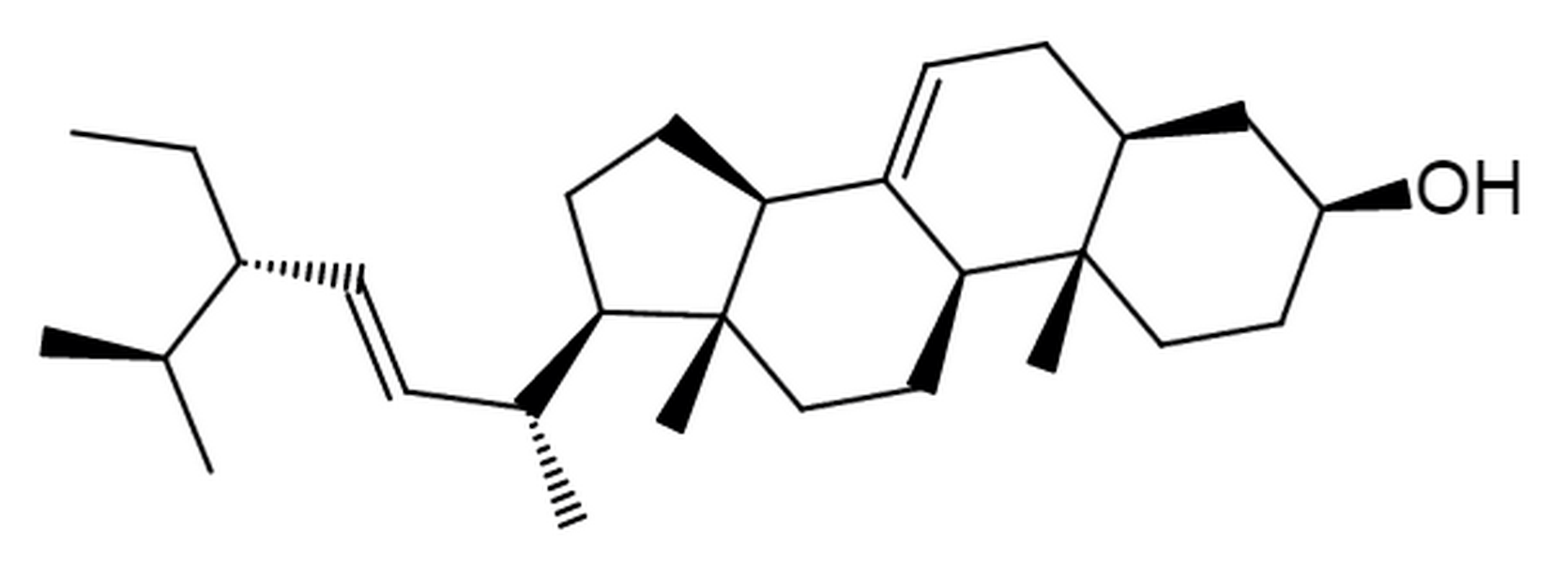 |
| [9064](https://pubchem.ncbi.nlm.nih.gov/compound/9064) | (+)-catechin | [C15H14O6](https://pubchem.ncbi.nlm.nih.gov/search/#query=C15H14O6) | 54.83 | 0.24 | 3 | 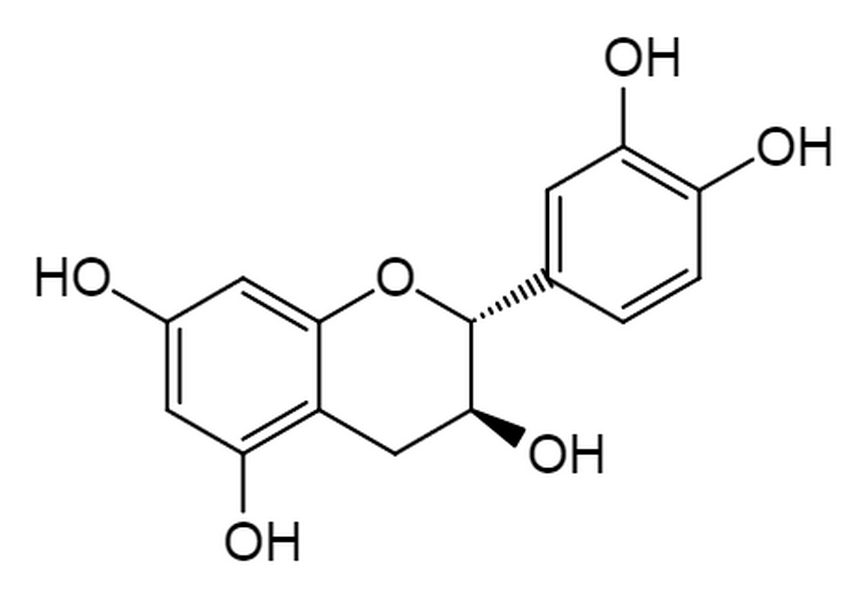 |
| [119307](https://pubchem.ncbi.nlm.nih.gov/compound/119307) | ginsenoside rh2 | [C36H62O8](https://pubchem.ncbi.nlm.nih.gov/search/#query=C36H62O8) | 36.32 | 0.56 | 3 | 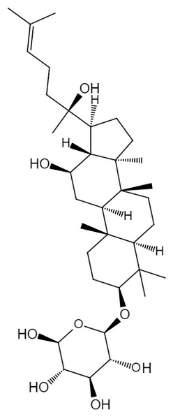 |
| [5281330](https://pubchem.ncbi.nlm.nih.gov/compound/5281330) | Poriferasterol | [C29H48O](https://pubchem.ncbi.nlm.nih.gov/search/#query=C29H48O) | 43.83 | 0.76 | 2 | 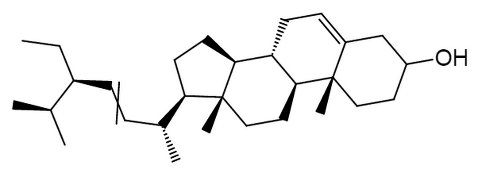 |
| 10100906 | Delphin_qt | C27H31ClO17 | 57.76 | 0.28 | 2 | 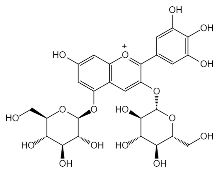 |
| [21679042](https://pubchem.ncbi.nlm.nih.gov/compound/21679042) | Deoxyandrographolide | [C20H30O4](https://pubchem.ncbi.nlm.nih.gov/search/#query=C20H30O4) | 56.3 | 0.31 | 2 | 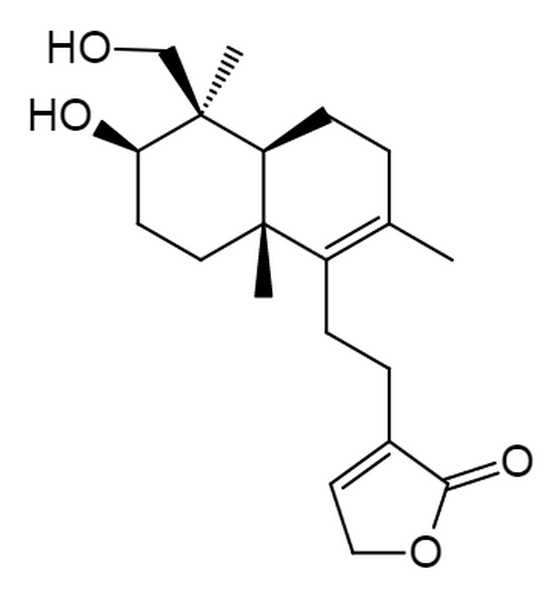 |
| [15976101](https://pubchem.ncbi.nlm.nih.gov/compound/15976101) | CID15976101 | [C30H52O](https://pubchem.ncbi.nlm.nih.gov/search/#query=C30H52O) | 36.23 | 0.78 | 2 | 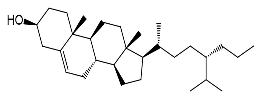 |
| [108213](https://pubchem.ncbi.nlm.nih.gov/compound/108213) | Bifendate | [C20H18O10](https://pubchem.ncbi.nlm.nih.gov/search/#query=C20H18O10) | 31.1 | 0.67 | 2 | 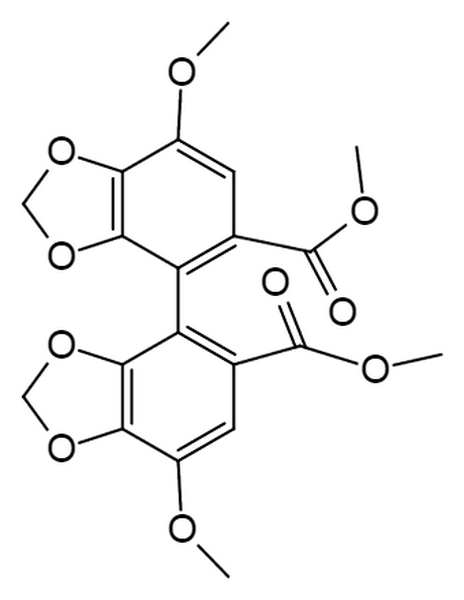 |
| 6037 | FA | C19H19N7O6 | 68.96 | 0.71 | 2 |  |
| 5316760 | CID5316760 | C17H14O6 | 39.05 | 0.48 | 2 | 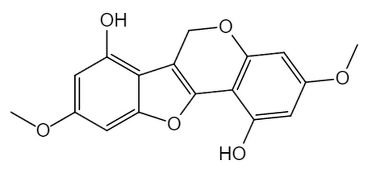 |
| [443758](https://pubchem.ncbi.nlm.nih.gov/compound/443758) | cis-Dihydroquercetin | [C15H12O7](https://pubchem.ncbi.nlm.nih.gov/search/#query=C15H12O7) | 66.44 | 0.27 | 2 | 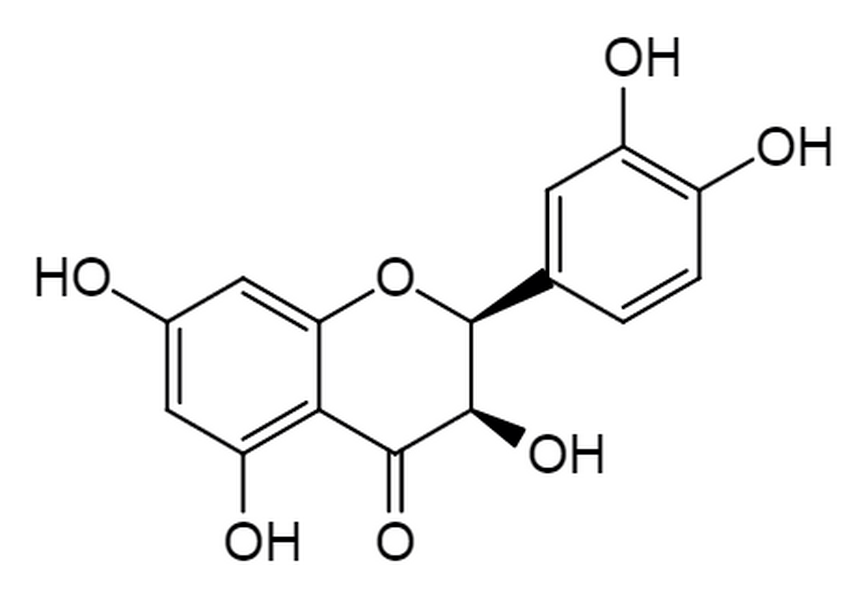 |
| [5870](https://pubchem.ncbi.nlm.nih.gov/compound/5870) | estrone | [C18H22O2](https://pubchem.ncbi.nlm.nih.gov/search/#query=C18H22O2) | 53.56 | 0.32 | 2 | 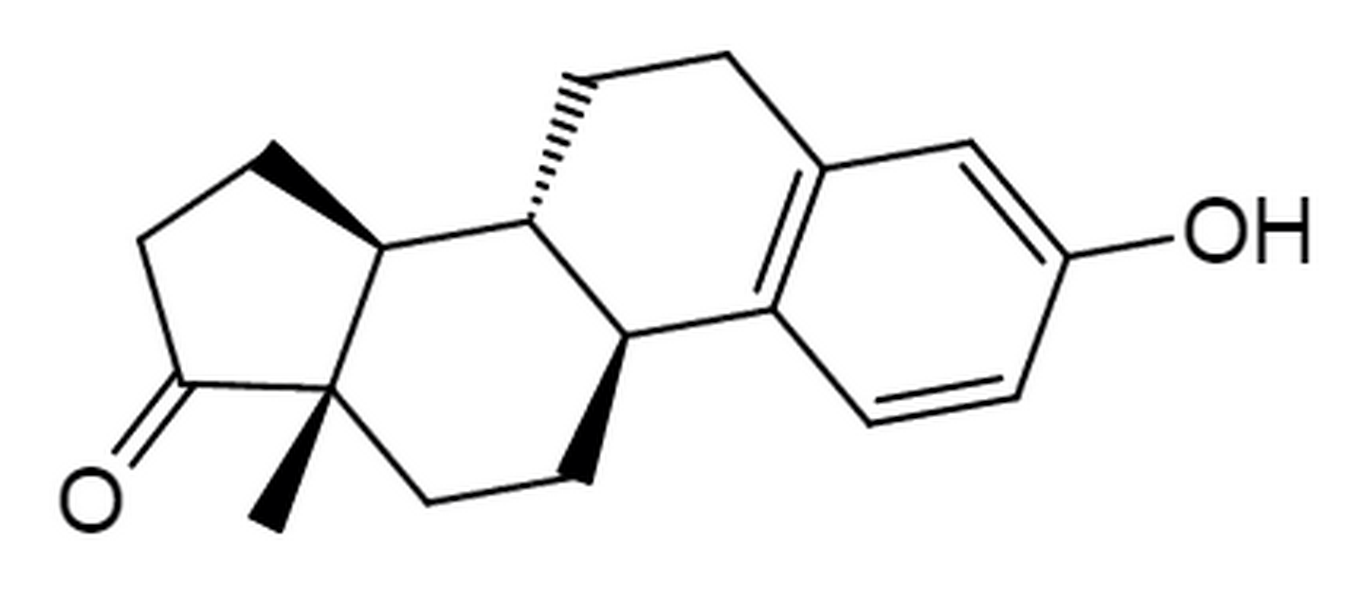 |
| [5282768](https://pubchem.ncbi.nlm.nih.gov/compound/5282768) | gondoic acid | [C20H38O2](https://pubchem.ncbi.nlm.nih.gov/search/#query=C20H38O2) | 30.7 | 0.2 | 2 | 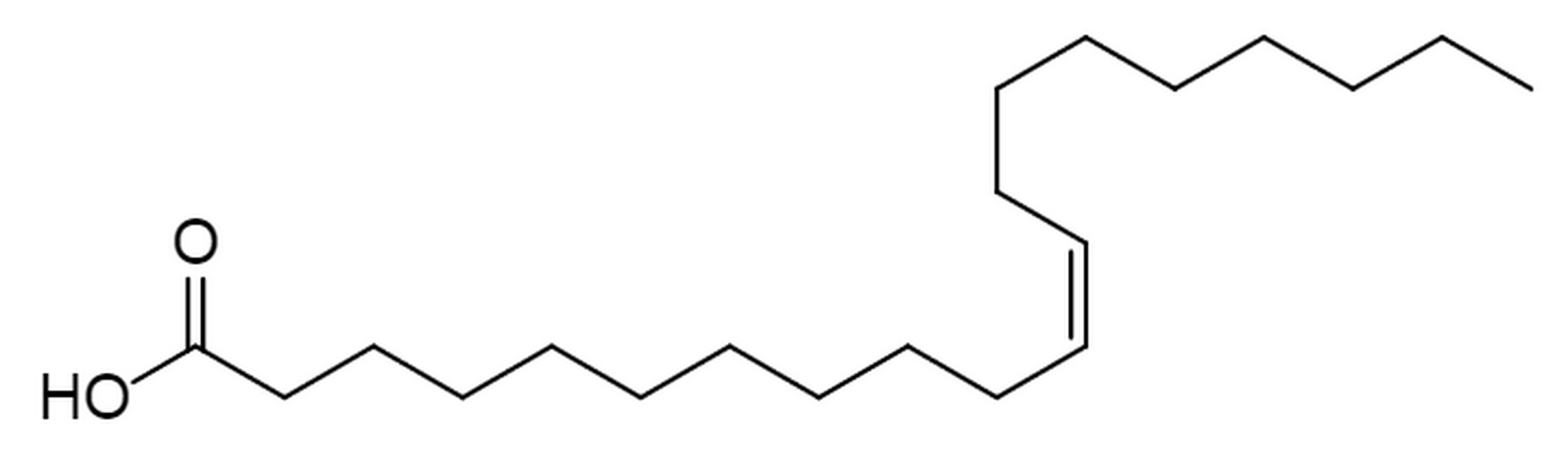 |
| 5997 | CLR | C27H46O | 37.87 | 0.68 | 2 | 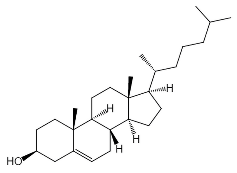 |
| [281691](https://pubchem.ncbi.nlm.nih.gov/compound/281691) | Machiline | [C17H19NO3](https://pubchem.ncbi.nlm.nih.gov/search/#query=C17H19NO3) | 79.64 | 0.24 | 2 | 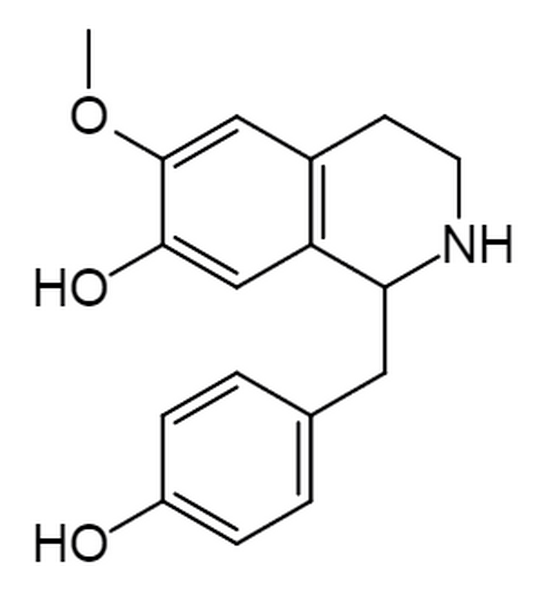 |
| [6917970](https://pubchem.ncbi.nlm.nih.gov/compound/6917970) | l-SPD | [C19H21NO4](https://pubchem.ncbi.nlm.nih.gov/search/#query=C19H21NO4) | 87.35 | 0.54 | 2 | 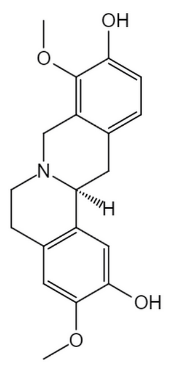 |
| [5283669](https://pubchem.ncbi.nlm.nih.gov/compound/5283669) | campesta-7,22E-dien-3beta-ol | [C28H46O](https://pubchem.ncbi.nlm.nih.gov/search/#query=C28H46O) | 43.51 | 0.72 | 2 | 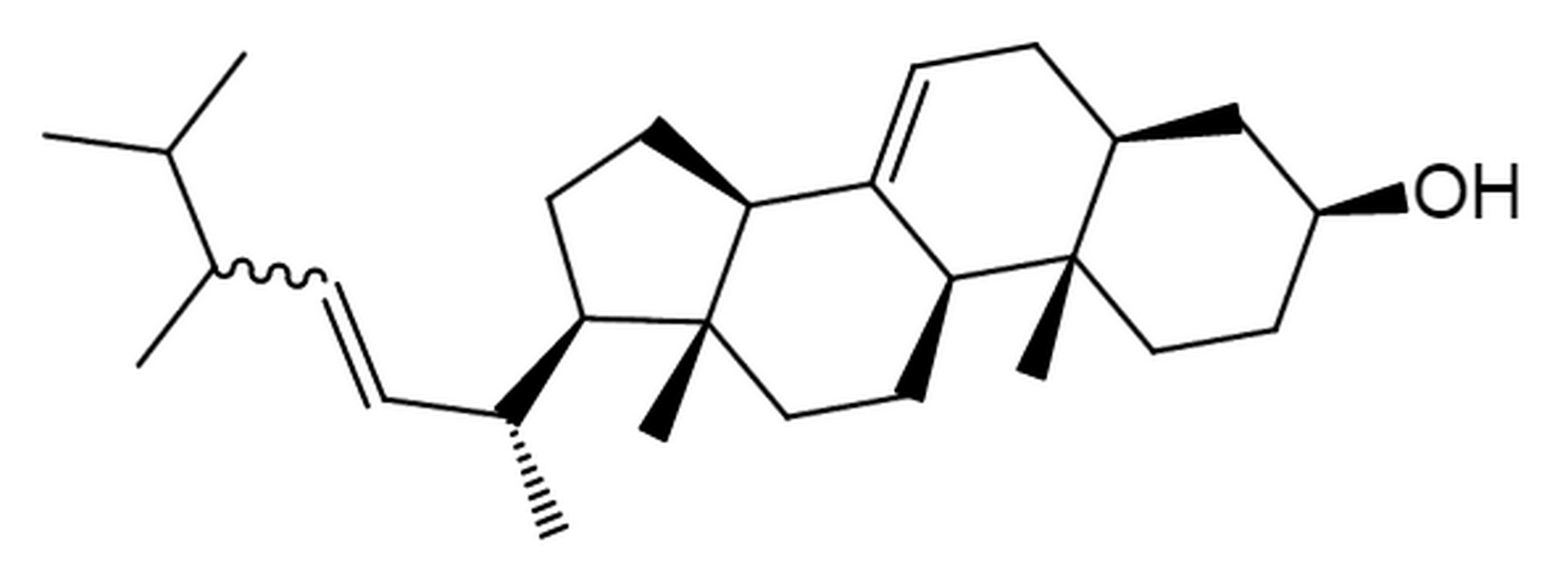 |
| [56676695](https://pubchem.ncbi.nlm.nih.gov/compound/56676695) | ergosta-4,6,8(14),22-tetraene-3-one | [C28H40O](https://pubchem.ncbi.nlm.nih.gov/search/#query=C28H40O) | 48.32 | 0.75 | 2 | 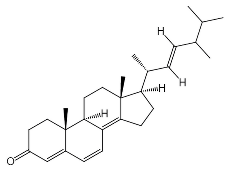 |
| [14015440](https://pubchem.ncbi.nlm.nih.gov/compound/14015440) | ganoderal B | [C30H46O3](https://pubchem.ncbi.nlm.nih.gov/search/#query=C30H46O3) | 42.56 | 0.81 | 2 | 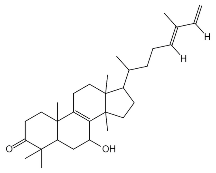 |
| [10343868](https://pubchem.ncbi.nlm.nih.gov/compound/10343868) | Lucialdehyde B | [C30H44O3](https://pubchem.ncbi.nlm.nih.gov/search/#query=C30H44O3) | 43.12 | 0.81 | 2 | 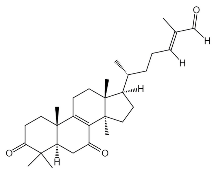 |
| [5283628](https://pubchem.ncbi.nlm.nih.gov/compound/5283628) | ergosta-7,22E-dien-3beta-ol | [C28H46O](https://pubchem.ncbi.nlm.nih.gov/search/#query=C28H46O) | 43.51 | 0.72 | 2 | 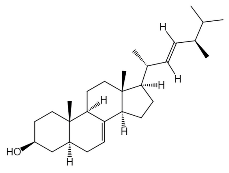 |
| 12444466 | Tremulone | C29H46O | 43.87 | 0.75 | 2 | 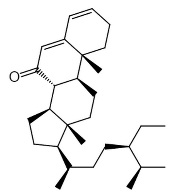 |
| [441837](https://pubchem.ncbi.nlm.nih.gov/compound/441837) | stigmast-7-enol | [C29H50O](https://pubchem.ncbi.nlm.nih.gov/search/#query=C29H50O) | 37.42 | 0.75 | 2 | 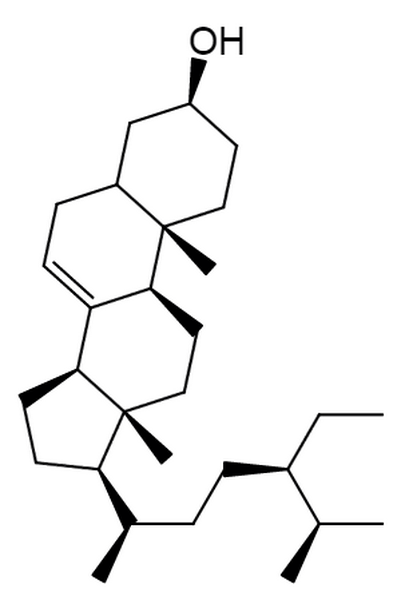 |
| [4680](https://pubchem.ncbi.nlm.nih.gov/compound/4680) | papaverine | [C20H21NO4](https://pubchem.ncbi.nlm.nih.gov/search/#query=C20H21NO4) | 64.04 | 0.38 | 2 | 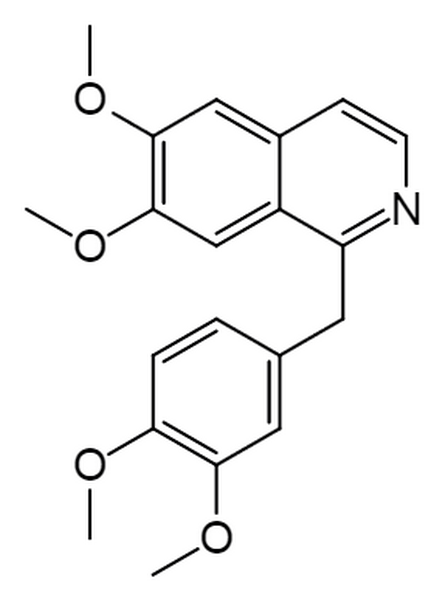 |
| 5742590 | daucosterol_qt | C35H60O6 | 36.91 | 0.75 | 2 | 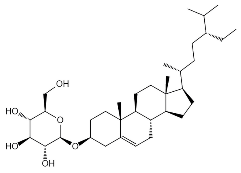 |
